# Supplementary material for: Estimating multiplicity of infection, allele frequencies, and prevalences accounting for incomplete data
Source: PLoS One. 2024 Mar 21;19(3):e0287161. doi: 10.1371/journal.pone.0287161 (PMC10956774; doi:10.1371/journal.pone.0287161)
Supplement: S1 Appendix — (PDF) [file pone.0287161.s001.pdf]

S1 Mathematical appendix

Model deviations

The probability distribution of incomplete observations

Accounting for incomplete data, equation (7a) is the explicit formula for the probability of observing $\boldsymbol{x}$. Here, we show the derivation of (7b) from (7a) in detail. For a non-empty record, $\boldsymbol{x} \neq \boldsymbol{0}$ we have

$$\begin{aligned}\tilde{Q}_{\boldsymbol{x}} &= \sum_{\boldsymbol{x} \leq \boldsymbol{y}} Q_{\boldsymbol{y}} \prod_{k=1}^{n} (1-\varepsilon)^{x_k} \varepsilon^{y_k - x_k} \\ &= (1-\varepsilon)^{|\boldsymbol{x}|} \sum_{\boldsymbol{x} \leq \boldsymbol{y}} Q_{\boldsymbol{y}} \prod_{k=1}^{n} \varepsilon^{y_k - x_k} \\ &= (1-\varepsilon)^{|\boldsymbol{x}|} Q_{\boldsymbol{x}} \sum_{\boldsymbol{x} \leq \boldsymbol{y}} \prod_{k=1}^{n} \Big(\varepsilon\big(e^{\lambda p_k} - 1\big)\Big)^{y_k - x_k},\end{aligned} \tag{1}$$

where $|\boldsymbol{x}| = \sum_{k=1}^{n} x_k$. By defining $\mathcal{A}(\boldsymbol{x}) = \{k \,|\, x_k = 0\}$, the above can be rewritten as

$$\tilde{Q}_{\boldsymbol{x}} = (1-\varepsilon)^{|\boldsymbol{x}|} Q_{\boldsymbol{x}} \sum_{\mathcal{F} \subseteq \mathcal{A}(\boldsymbol{x})} \prod_{h \in \mathcal{F}} \Big(\varepsilon\big(e^{\lambda p_h} - 1\big)\Big). \tag{2}$$

Note, for a set of numbers $a_1, \ldots, a_n$ we have

$$\prod_{k=1}^{n} (a_k + 1) = \sum_{\mathcal{F} \subseteq \{1,\ldots,n\}} \prod_{h \in \mathcal{F}} a_h. \tag{3}$$

This identity can be used to write (1) as

$$\tilde{Q}_{\boldsymbol{x}} = (1-\varepsilon)^{|\boldsymbol{x}|} Q_{\boldsymbol{x}} \prod_{h \in \mathcal{A}(\boldsymbol{x})} \Big(\varepsilon\big(e^{\lambda p_h} - 1\big) + 1\Big) = (1-\varepsilon)^{|\boldsymbol{x}|} Q_{\boldsymbol{x}} \prod_{k=1}^{n} \Big(\varepsilon\big(e^{\lambda p_k} - 1\big) + 1\Big)^{1-x_k}. \tag{4}$$

By replacing $Q_{\boldsymbol{x}}$ with its equivalent given in (4b), we derive

$$\tilde{Q}_{\boldsymbol{x}} = \frac{1}{e^{\lambda} - 1} \prod_{k=1}^{n} \Big((1-\varepsilon)\big(e^{\lambda p_k} - 1\big)\Big)^{x_k} \Big(\varepsilon\big(e^{\lambda p_k} - 1\big) + 1\Big)^{1-x_k}. \tag{5}$$

The empty record $\boldsymbol{x} = \boldsymbol{0} = (0, \ldots, 0)$ results from a true infection, which contains at least one lineage, i.e., $\boldsymbol{0} < \boldsymbol{y}$. Hence,

$$\tilde{Q}_{\boldsymbol{0}} = \sum_{\boldsymbol{0} < \boldsymbol{y}} Q_{\boldsymbol{y}} \prod_{k=1}^{n} \varepsilon^{y_k}. \tag{6}$$

By formally defining $Q_{\boldsymbol{0}} := \dfrac{1}{e^{\lambda} - 1}$, and noticing that $\mathcal{A}(\boldsymbol{0}) = \{1, \ldots, n\}$, we derive

$$\begin{aligned}\tilde{Q}_{\boldsymbol{0}} &= -\frac{1}{e^{\lambda} - 1} + \sum_{\boldsymbol{0} \leq \boldsymbol{y}} Q_{\boldsymbol{y}} \prod_{k=1}^{n} \varepsilon^{y_k} \\ &= \frac{1}{e^{\lambda} - 1} \left(-1 + \sum_{\boldsymbol{0} \leq \boldsymbol{y}} \prod_{k=1}^{n} \Big(\varepsilon\big(e^{\lambda p_k} - 1\big)\Big)^{y_k} \right) \\ &= \frac{1}{e^{\lambda} - 1} \left(-1 + \sum_{\mathcal{F} \subseteq \mathcal{A}(\boldsymbol{x})} \prod_{h \in \mathcal{F}} \Big(\varepsilon\big(e^{\lambda p_h} - 1\big)\Big) \right).\end{aligned}$$

By using the identity in (3), the probability of observing the empty record becomes

$$\tilde{Q}_{\mathbf{0}} = \frac{1}{e^{\lambda} - 1}\left(- 1 + \prod_{k=1}^{n} \left(\varepsilon(e^{\lambda p_k} - 1) + 1\right)\right). \tag{8}$$

Prevalence

Next follows the proof of Remark 1.

Proof of Remark 1. Without loss of generality, we derive the observable prevalence of lineage A_n. As $\tilde{Q}_{\boldsymbol{x}}$ is the probability mass function of $\boldsymbol{x}$, we have

$$1 = \sum_{\boldsymbol{x}\in\tilde{\mathcal{O}}} \tilde{Q}_{\boldsymbol{x}} = \sum_{\substack{\boldsymbol{x}\in\tilde{\mathcal{O}}:\\ x_n=0}} \tilde{Q}_{\boldsymbol{x}} + \sum_{\substack{\boldsymbol{x}\in\tilde{\mathcal{O}}:\\ x_n=1}} \tilde{Q}_{\boldsymbol{x}}. \tag{9}$$

The second sum on the right-hand side runs over all configurations with $x_n = 1$, and hence, is the prevalence of lineage A_n, which we denote by $\tilde{q}_n$. Let $\boldsymbol{e}_n = (0, \dots, 0, 1)$ be the configuration corresponding to observing only lineage A_n in an infection. According to (5), the probability of configuration $\boldsymbol{x} = \boldsymbol{e}_n$ is

$$\tilde{Q}_{\boldsymbol{e}_n} = \frac{1}{e^{\lambda} - 1}\Big((1-\varepsilon)(e^{\lambda p_n} - 1)\Big)\prod_{k=1}^{n-1} \left(\varepsilon(e^{\lambda p_k} - 1) + 1\right). \tag{10}$$

Further, rearrangement of (9) gives

$$1 - \tilde{q}_n = \sum_{\substack{\boldsymbol{x}\in\tilde{\mathcal{O}}:\\ x_n=0}} \tilde{Q}_{\boldsymbol{x}} = \tilde{Q}_{\mathbf{0}} + \sum_{\substack{\boldsymbol{x}\in\tilde{\mathcal{O}}\setminus\{\mathbf{0}\}:\\ x_n=0}} \tilde{Q}_{\boldsymbol{x}}.$$

By using (5), we realize that the sum on the right-hand side can be rewritten, so that the above equation becomes

$$1 - \tilde{q}_n = \tilde{Q}_{\mathbf{0}} + \frac{\varepsilon(e^{\lambda p_n} - 1) + 1}{(1-\varepsilon)(e^{\lambda p_n} - 1)} \sum_{\substack{\boldsymbol{x}\in\tilde{\mathcal{O}}\setminus\{\boldsymbol{e}_n\}:\\ x_n=1}} \tilde{Q}_{\boldsymbol{x}} \tag{11}$$

$$= \tilde{Q}_{\mathbf{0}} + \frac{\varepsilon(e^{\lambda p_n} - 1) + 1}{(1-\varepsilon)(e^{\lambda p_n} - 1)}\Big(\sum_{\substack{\boldsymbol{x}\in\tilde{\mathcal{O}}:\\ x_n=1}} \tilde{Q}_{\boldsymbol{x}} - \tilde{Q}_{\boldsymbol{e}_n}\Big) \tag{12}$$

$$= \tilde{Q}_{\mathbf{0}} + \frac{\varepsilon(e^{\lambda p_n} - 1) + 1}{(1-\varepsilon)(e^{\lambda p_n} - 1)}\tilde{q}_n - \frac{1}{e^{\lambda} - 1}\prod_{k=1}^{n} \left(\varepsilon(e^{\lambda p_k} - 1) + 1\right). \tag{13}$$

Because $\tilde{Q}_{\mathbf{0}}$ is given by (8) we further obtain

$$1 - \tilde{q}_n = \frac{\varepsilon(e^{\lambda p_n} - 1) + 1}{(1-\varepsilon)(e^{\lambda p_n} - 1)}\tilde{q}_n - \frac{1}{e^{\lambda} - 1},$$

which after some algebraic manipulations yields

$$\tilde{q}_n = \sum_{\substack{\boldsymbol{x}\in\tilde{\mathcal{O}}:\\ x_n=1}} \tilde{Q}_{\boldsymbol{x}} = (1-\varepsilon)\frac{1 - e^{-\lambda p_n}}{1 - e^{-\lambda}}. \tag{14}$$

The observable prevalence of lineage A_n, $\tilde{q}_n$, is the probability that A_n is actually observed in a sample. Since the empty records do not provide any information concerning the lineages present the corresponding infections, it might be more

convenient to not account for them in the definition of prevalence, i.e., to condition the observable prevalence on not observing the non-empty records, i.e., $\boldsymbol{x} \neq \mathbf{0}$. The conditional prevalence of lineage A_n is

$$\tilde{q}_n^{(c)} = \frac{\tilde{q}_n}{1 - \tilde{Q}_{\mathbf{0}}} = \frac{(1-\varepsilon)(1-e^{-\lambda p_n})}{1 - \prod_{k=1}^{n}\Big(\varepsilon(1-e^{-\lambda p_k}) + e^{-\lambda p_k}\Big)}. \tag{15}$$

Since n was arbitrary, it can be replaced by any $k = 1, \ldots, n-1$. □

Super-infections rarely occur if the MOI parameter λ is close to zero. Considering the limit case yields

$$\lim_{\lambda \to 0} q_k = \lim_{\lambda \to 0} \frac{(1-\varepsilon)(1-e^{-\lambda p_k})}{1 - \prod_{l=1}^{n}\Big(\varepsilon(1-e^{-\lambda p_l}) + e^{-\lambda p_l}\Big)}. \tag{16}$$

The nominator and denominator of the right-hand side of (16) both equal zero for $\lambda = 0$. Application of l'Hospital's rule gives

$$\lim_{\lambda \to 0} q_k = \lim_{\lambda \to 0} \frac{(1-\varepsilon)p_k e^{-\lambda p_k}}{\prod_{l=1}^{n}\Big(\varepsilon(1-e^{-\lambda p_l}) + e^{-\lambda p_l}\Big)\sum_{l=1}^{n}\frac{p_l(e^{-\lambda p_l} - \varepsilon e^{-\lambda p_l})}{\varepsilon(1-e^{-\lambda p_l}) + e^{-\lambda p_l}}} = \frac{(1-\varepsilon)p_k}{1-\varepsilon} = p_k. \tag{17}$$

Hence, if the MOI parameter rate is close to zero, a lineage's prevalence is close to its frequency.

Log-likelihood function

Under the IDM, the log-likelihood function corresponding to the observed dataset $\mathcal{X}$ consisting of N independent observations $\boldsymbol{x}^{(1)}, \ldots, \boldsymbol{x}^{(N)}$ is given by equation (13). Now, we simplify the log-likelihood function. Remember, we defined $\tilde{\mathcal{O}} = \{0,1\}^n$ and $\mathcal{O} = \{0,1\}^n \backslash \{\mathbf{0}\}$, and $n_{\boldsymbol{x}}$ as the number of samples in $\mathcal{X}$ with configuration $\boldsymbol{x}$. In particular, $n_{\mathbf{0}}$ was defined as the number of empty records in $\mathcal{X}$. The log-likelihood function becomes

$$\ell(\boldsymbol{\theta}) = \sum_{\boldsymbol{x} \in \tilde{\mathcal{O}}} n_{\boldsymbol{x}} \log \tilde{Q}_{\boldsymbol{x}} = n_{\mathbf{0}} \log \tilde{Q}_{\mathbf{0}} + \sum_{\boldsymbol{x} \in \mathcal{O}} n_{\boldsymbol{x}} \log \tilde{Q}_{\boldsymbol{x}}. \tag{18}$$

The first and second terms on the right-hand side of (18) are derived respectively as

$$n_{\mathbf{0}} \log \tilde{Q}_{\mathbf{0}} = n_{\mathbf{0}}\left(-\log\Big(e^{\lambda} - 1\Big) + \log\left(-1 + \prod_{k=1}^{n}\Big(\varepsilon(e^{\lambda p_k} - 1) + 1\Big)\right)\right) \tag{19a}$$

and

$$\begin{aligned}\sum_{\boldsymbol{x} \in \mathcal{O}} n_{\boldsymbol{x}} \log \tilde{Q}_{\boldsymbol{x}} &= \sum_{\boldsymbol{x} \in \mathcal{O}} n_{\boldsymbol{x}}\left(-\log(e^{\lambda} - 1) + \sum_{k=1}^{n}\left(\log(1-\varepsilon)x_k + x_k \log(e^{\lambda p_k} - 1)\right.\right.\\ &\qquad \left.\left. + (1-x_k)\log\Big(\varepsilon(e^{\lambda p_k} - 1) + 1\Big)\right)\right)\\ &= -\sum_{\boldsymbol{x} \in \mathcal{O}} n_{\boldsymbol{x}} \log(e^{\lambda} - 1) \\ &\quad + \sum_{k=1}^{n}\left(\log(1-\varepsilon)\sum_{\boldsymbol{x} \in \mathcal{O}} x_k n_{\boldsymbol{x}} + \log(e^{\lambda p_k} - 1)\sum_{\boldsymbol{x} \in \mathcal{O}} x_k n_{\boldsymbol{x}}\right.\\ &\qquad \left. + \log\Big(\varepsilon(e^{\lambda p_k} - 1) + 1\Big)\sum_{\boldsymbol{x} \in \mathcal{O}}(1-x_k)n_{\boldsymbol{x}}\right).\end{aligned} \tag{19b}$$

To simplify (19b), note that the number of samples in the dataset $\mathcal{X}$ for which lineage A_k is observed was denoted by N_k (14). Clearly,

$$N_k = \sum_{\boldsymbol{x}\in\tilde{\mathcal{O}}} x_k n_{\boldsymbol{x}} = \sum_{\boldsymbol{x}\in\mathcal{O}} x_k n_{\boldsymbol{x}}.$$

Consequently, we have

$$N_+ - N_k = \sum_{\boldsymbol{x}\in\mathcal{O}} (1 - x_k) n_{\boldsymbol{x}},$$

where N_+ was defined as the number of non-empty records, which in mathematical notation is (cf. eq. 16)

$$N_+ = \sum_{\boldsymbol{x}\in\mathcal{O}} n_{\boldsymbol{x}} = \Big(\sum_{\boldsymbol{x}\in\tilde{\mathcal{O}}} n_{\boldsymbol{x}}\Big) - n_{\mathbf{0}} = N - n_{\mathbf{0}}.$$

Hence, (19b) can be rewritten as

$$\begin{aligned}\sum_{\boldsymbol{x}\in\mathcal{O}} n_{\boldsymbol{x}} \log \tilde{Q}_{\boldsymbol{x}} = &- N_+ \log(e^{\lambda} - 1) + \log(1-\varepsilon) \sum_{k=1}^{n} N_k \\ &+ \sum_{k=1}^{n} N_k \log(e^{\lambda p_k} - 1) + \sum_{k=1}^{n} (N_+ - N_k) \log\Big(\varepsilon(e^{\lambda p_k} - 1) + 1\Big).\end{aligned}$$

By noting that $N = n_{\mathbf{0}} + N_+$, the log-likelihood function is derived as

$$\begin{aligned}\ell(\boldsymbol{\theta}) = &- N_+ \log(e^{\lambda} - 1) + \log(1-\varepsilon) \sum_{k=1}^{n} N_k + \sum_{k=1}^{n} N_k \log(e^{\lambda p_k} - 1) \\ &+ \sum_{k=1}^{n} (N_+ - N_k) \log\Big(\varepsilon(e^{\lambda p_k} - 1) + 1\Big) \\ &+ n_{\mathbf{0}}\left(- \log(e^{\lambda} - 1) + \log\left(-1 + \prod_{k=1}^{n} \Big(\varepsilon(e^{\lambda p_k} - 1) + 1\Big)\right)\right) \\ = &- N \log(e^{\lambda} - 1) + \log(1-\varepsilon) \sum_{k=1}^{n} N_k + \sum_{k=1}^{n} N_k \log(e^{\lambda p_k} - 1) \\ &+ \sum_{k=1}^{n} (N - N_k) \log\Big(\varepsilon(e^{\lambda p_k} - 1) + 1\Big) \\ &+ n_{\mathbf{0}} \log\left(1 - \frac{1}{\prod_{k=1}^{n} \Big(\varepsilon(e^{\lambda p_k} - 1) + 1\Big)}\right).\end{aligned}$$

Inverse Fisher information

Because the frequency vector $\boldsymbol{p}$ is an element of the $n-1$ dimensional simplex, it is convenient to introduce a Lagrange multiplier to maximize the likelihood function (21). Then the resulting score function needs to be equated to zero. Unfortunately, no closed solution exists for the MLE. Hence, it needs to be derived numerically. A straightforward, but numerically not advisable, approach would be to use a Newton method. This would require all second-order partial derivatives of the (21). The derivation of these partial derivatives is presented here, not merely because of completeness, but because it is convenient to derive the inverse Fisher information from these second derivatives, which retain the Lagrange multiplier as a nuisance parameter (cf. [1]).

To simplify the notation, let

$$\tau = \prod_{l=1}^{n} \Big(\varepsilon (e^{\lambda p_l} - 1) + 1 \Big). \tag{20}$$

For $k = 1, \dots, n$, the first partial derivatives of the Lagrange function (21) with respect to the model parameters are calculated to be

$$\begin{aligned} \frac{\partial \Lambda}{\partial \lambda} =& \frac{\partial \ell}{\partial \lambda} = \sum_{l=1}^{n} N_l \frac{p_l e^{\lambda p_l}}{e^{\lambda p_l} - 1} + \sum_{l=1}^{n} (N - N_l) \frac{\varepsilon p_l e^{\lambda p_l}}{\varepsilon (e^{\lambda p_l} - 1) + 1} + \frac{n_{\mathbf{0}} \varepsilon}{\tau - 1} \sum_{l=1}^{n} \frac{p_l e^{\lambda p_l}}{\varepsilon (e^{\lambda p_l} - 1) + 1} \\ & - \frac{N}{1 - e^{-\lambda}}, \end{aligned} \tag{21a}$$

$$\frac{\partial \Lambda}{\partial \varepsilon} = \frac{\partial \ell}{\partial \varepsilon} = -\frac{1}{1 - \varepsilon} \sum_{l=1}^{n} N_l + \sum_{l=1}^{n} (N - N_l) \frac{e^{\lambda p_l} - 1}{\varepsilon (e^{\lambda p_l} - 1) + 1} + \frac{n_{\mathbf{0}}}{\tau - 1} \sum_{l=1}^{n} \frac{e^{\lambda p_l} - 1}{\varepsilon (e^{\lambda p_l} - 1) + 1}, \tag{21b}$$

$$\frac{\partial \Lambda}{\partial p_k} = \frac{\partial \ell}{\partial p_k} = N_k \frac{\lambda e^{\lambda p_k}}{e^{\lambda p_k} - 1} + (N - N_k) \frac{\varepsilon \lambda e^{\lambda p_k}}{\varepsilon (e^{\lambda p_k} - 1) + 1} + \frac{n_{\mathbf{0}}}{\tau - 1} \frac{\varepsilon \lambda e^{\lambda p_k}}{\varepsilon (e^{\lambda p_k} - 1) + 1} - \gamma, \tag{21c}$$

and

$$\frac{\partial \Lambda}{\partial \gamma} = 1 - \sum_{l=1}^{n} p_l. \tag{21d}$$

The second-order partial derivatives, i.e., the entries of the Hessian matrix of the

Lagrange function (21) are derived as

$$\begin{aligned}\frac{\partial^2\Lambda}{\partial\lambda^2} =&\frac{\partial^2\ell}{\partial\lambda^2}\\ =&N\frac{e^{-\lambda}}{(1-e^{-\lambda})^2}-\sum_{l=1}^{n}p_l^2\left(N_l\frac{e^{\lambda p_l}}{(e^{\lambda p_l}-1)^2}-(N-N_l)\frac{\varepsilon(1-\varepsilon)e^{\lambda p_l}}{\left(\varepsilon\left(e^{\lambda p_l}-1\right)+1\right)^2}\right)\\ &+\frac{n_{\mathbf{0}}}{\tau-1}\left(\varepsilon(1-\varepsilon)\sum_{l=1}^{n}\frac{p_l^2e^{\lambda p_l}}{\left(\varepsilon\left(e^{\lambda p_l}-1\right)+1\right)^2}+\frac{\tau\varepsilon^2}{1-\tau}\left(\sum_{l=1}^{n}\frac{p_le^{\lambda p_l}}{\varepsilon(e^{\lambda p_l}-1)+1}\right)^2\right),\end{aligned} \tag{22a}$$

$$\begin{aligned}\frac{\partial^2\Lambda}{\partial\lambda\partial\varepsilon} =&\frac{\partial^2\ell}{\partial\lambda\partial\varepsilon}\\ =&\sum_{l=1}^{n}(N-N_l)\frac{p_le^{\lambda p_l}}{\left(\varepsilon\left(e^{\lambda p_l}-1\right)+1\right)^2}\\ &+\frac{n_{\mathbf{0}}}{\tau-1}\left(\frac{\tau}{1-\tau}\left(\sum_{l=1}^{n}\frac{e^{\lambda p_l}-1}{\varepsilon(e^{\lambda p_l}-1)+1}\right)\left(\varepsilon\sum_{l=1}^{n}\frac{p_le^{\lambda p_l}}{\varepsilon(e^{\lambda p_l}-1)+1}\right)\right.\\ &\left.+\sum_{l=1}^{n}\frac{p_le^{\lambda p_l}}{(\varepsilon(e^{\lambda p_l}-1)+1)^2}\right),\end{aligned} \tag{22b}$$

$$\begin{aligned}\frac{\partial^2\Lambda}{\partial\lambda\partial p_k} =&\frac{\partial^2\ell}{\partial\lambda\partial p_k}\\ =&e^{\lambda p_k}\left(N_k\frac{e^{\lambda p_k}-1-\lambda p_k}{(e^{\lambda p_k}-1)^2}+(N-N_k)\frac{\varepsilon\left(\varepsilon e^{\lambda p_k}+(1-\varepsilon)(1+\lambda p_k)\right)}{\left(\varepsilon\left(e^{\lambda p_k}-1\right)+1\right)^2}\right)\\ &+\frac{n_{\mathbf{0}}}{\tau-1}\frac{\varepsilon e^{\lambda p_k}}{\varepsilon(e^{\lambda p_k}-1)+1}\left(\frac{\varepsilon e^{\lambda p_k}+(1-\varepsilon)(1+\lambda p_k)}{\varepsilon(e^{\lambda p_k}-1)+1}\right.\\ &\left.+\frac{\tau\lambda\varepsilon}{1-\tau}\sum_{l=1}^{n}\frac{p_le^{\lambda p_l}}{\varepsilon(e^{\lambda p_l}-1)+1}\right),\end{aligned} \tag{22c}$$

$$\frac{\partial^2 \Lambda}{\partial \varepsilon^2} = \frac{\partial^2 \ell}{\partial \varepsilon^2} = -\frac{1}{(1-\varepsilon)^2}\sum_{l=1}^{n} N_l - \sum_{l=1}^{n}(N-N_l)\left(\frac{e^{\lambda p_l}-1}{\varepsilon(e^{\lambda p_l}-1)+1}\right)^2 - \frac{n_{\mathbf{0}}}{\tau-1}\left(\frac{\tau}{\tau-1}\left(\sum_{l=1}^{n}\frac{e^{\lambda p_l}-1}{\varepsilon(e^{\lambda p_l}-1)+1}\right)^2 + \sum_{l=1}^{n}\left(\frac{e^{\lambda p_l}-1}{\varepsilon(e^{\lambda p_l}-1)+1}\right)^2\right), \tag{22d}$$

$$\begin{aligned}\frac{\partial^2 \Lambda}{\partial \varepsilon \partial p_k} =& \frac{\partial^2 \ell}{\partial \varepsilon \partial p_k} \\ =& (N-N_k)\frac{\lambda e^{\lambda p_k}}{(\varepsilon(e^{\lambda p_k}-1)+1)^2} \\ &+ \frac{n_{\mathbf{0}}}{\tau-1}\frac{\lambda e^{\lambda p_k}}{\varepsilon(e^{\lambda p_k}-1)+1}\left(\frac{1}{\varepsilon(e^{\lambda p_k}-1)+1} + \frac{\tau\varepsilon}{1-\tau}\sum_{l=1}^{n}\frac{e^{\lambda p_l}-1}{\varepsilon(e^{\lambda p_l}-1)+1}\right),\end{aligned} \tag{22e}$$

$$\frac{\partial^2 \Lambda}{\partial p_k^2} = \frac{\partial^2 \ell}{\partial p_k^2} = \lambda^2 e^{\lambda p_k}\left(-N_k\frac{1}{(e^{\lambda p_k}-1)^2} + (N-N_k)\frac{\varepsilon(1-\varepsilon)}{\left(\varepsilon(e^{\lambda p_k}-1)+1\right)^2}\right) + \frac{n_{\mathbf{0}}}{\tau-1}\frac{\lambda^2\varepsilon e^{\lambda p_k}}{\left(\varepsilon(e^{\lambda p_k}-1)+1\right)^2}\left(1-\varepsilon+\frac{\tau\varepsilon e^{\lambda p_k}}{1-\tau}\right), \tag{22f}$$

$$\frac{\partial^2 \Lambda}{\partial p_k \partial \gamma} = -1, \tag{22g}$$

$$\frac{\partial^2 \Lambda}{\partial \lambda \partial \gamma} = \frac{\partial^2 \Lambda}{\partial \varepsilon \partial \gamma} = \frac{\partial^2 \Lambda}{\partial \gamma^2} = 0, \tag{22h}$$

and

$$\frac{\partial^2 \Lambda}{\partial p_k \partial p_j} = 0, \tag{22i}$$

for $k, j = 1, \ldots, n$ $(k \neq j)$, where the order of the derivatives in (22) can be interchanged.

The Fisher information is derived by substituting (24) into (22) using Remark 1. After some algebraic manipulation, the entries of the Fisher information are

$$I_{\lambda,\lambda} = -\mathbb{E}\left(\frac{\partial^2 \Lambda}{\partial \lambda^2}\right) = \frac{-N}{e^\lambda - 1}\left(e^\lambda\left(\frac{1}{e^\lambda-1} - (1-\varepsilon)\sum_{l=1}^{n}\frac{p_l^2}{(e^{\lambda p_l}-1)\left(\varepsilon(e^{\lambda p_l}-1)+1\right)}\right) - \frac{\tau}{\tau-1}\left(\sum_{l=1}^{n}\frac{\varepsilon p_l e^{\lambda p_l}}{\varepsilon(e^{\lambda p_l}-1)+1}\right)^2\right), \tag{23a}$$

$$I_{\lambda,\varepsilon} = -\mathbb{E}\left(\frac{\partial^2 \Lambda}{\partial \lambda \partial \varepsilon}\right) = \frac{-N}{e^\lambda - 1}\left(e^\lambda \sum_{l=1}^{n}\frac{p_l}{\varepsilon(e^{\lambda p_l}-1)+1} - \frac{\tau}{\tau-1}\left(\sum_{l=1}^{n}\frac{\varepsilon p_l e^{\lambda p_l}}{\varepsilon(e^{\lambda p_l}-1)+1}\right)\left(\sum_{l=1}^{n}\frac{e^{\lambda p_l}-1}{\varepsilon(e^{\lambda p_l}-1)+1}\right)\right), \tag{23b}$$

$$I_{\lambda,p_k} = -\mathbb{E}\left(\frac{\partial^2 \Lambda}{\partial \lambda \partial p_k}\right) = \frac{-N}{e^\lambda - 1}\left(e^\lambda\left(1 - \frac{(1-\varepsilon)\lambda p_k}{(e^{\lambda p_k}-1)\left(\varepsilon(e^{\lambda p_k}-1)+1\right)}\right) - \frac{\tau}{\tau-1}\frac{\varepsilon\lambda e^{\lambda p_k}}{\varepsilon(e^{\lambda p_k}-1)+1}\left(\sum_{l=1}^{n}\frac{\varepsilon p_l e^{\lambda p_l}}{\varepsilon(e^{\lambda p_l}-1)+1}\right)\right), \tag{23c}$$

$$I_{\varepsilon,\varepsilon} = -\mathbb{E}\left(\frac{\partial^2\Lambda}{\partial\varepsilon^2}\right) = \frac{-N}{e^\lambda - 1}\left(e^\lambda\left(-\frac{1}{1-\varepsilon}\sum_{l=1}^{n}\frac{e^{\lambda p_l}-1}{\varepsilon(e^{\lambda p_l}-1)+1}\right) - \frac{\tau}{\tau-1}\left(\sum_{l=1}^{n}\frac{e^{\lambda p_l}-1}{\varepsilon(e^{\lambda p_l}-1)+1}\right)^2\right), \tag{23d}$$

$$I_{\varepsilon,p_k} = -\mathbb{E}\left(\frac{\partial^2\Lambda}{\partial\varepsilon\partial p_k}\right) = \frac{-N}{e^\lambda - 1}\left(e^\lambda\left(\frac{\lambda}{\varepsilon(e^{\lambda p_k}-1)+1}\right) - \frac{\tau}{\tau-1}\frac{\varepsilon\lambda e^{\lambda p_k}}{\varepsilon(e^{\lambda p_k}-1)+1}\sum_{l=1}^{n}\frac{e^{\lambda p_l}-1}{\varepsilon(e^{\lambda p_l}-1)+1}\right), \tag{23e}$$

$$I_{p_k,p_k} = -\mathbb{E}\left(\frac{\partial^2\Lambda}{\partial p_k^2}\right) = \frac{-N}{e^\lambda - 1}\left(e^\lambda\left(-\frac{(1-\varepsilon)\lambda^2}{(e^{\lambda p_k}-1)\big(\varepsilon(e^{\lambda p_k}-1)+1\big)}\right) - \frac{\tau}{\tau-1}\left(\frac{\varepsilon\lambda e^{\lambda p_k}}{\varepsilon(e^{\lambda p_k}-1)+1}\right)^2\right), \tag{23f}$$

$$I_{p_k,\gamma} = \mathbb{E}\left(-\frac{\partial^2\Lambda}{\partial p_k\partial\gamma}\right) = 1, \tag{23g}$$

$$I_{\lambda,\gamma} = I_{\varepsilon,\gamma} = I_{\gamma,\gamma} = -\mathbb{E}\left(\frac{\partial^2\Lambda}{\partial\lambda\partial\gamma}\right) = -\mathbb{E}\left(\frac{\partial^2\Lambda}{\partial\varepsilon\partial\gamma}\right) = -\mathbb{E}\left(\frac{\partial^2\Lambda}{\partial\gamma^2}\right) = 0, \tag{23h}$$

and

$$I_{p_k,p_j}\,\mathbb{E}\left(\frac{\partial^2\Lambda}{\partial p_k\partial p_j}\right) = 0, \tag{23i}$$

for $k, j = 1, \ldots, n$ $(k \neq j)$, where the entries are symmetric. Note that (i) the inverse Fisher information is of primary interest because it yields the asymptotic variance of the estimator and (ii) Lagrange-multiplier is kept as a nuisance parameter. Retaining the Lagrange-multiplier has the conceptional advantage that the structure of the Fisher information is simpler and hence it can be inverted more easily. A straightforward approach is block-wise inversion. Namely, the Fisher information has the following structure

$$\left(\begin{array}{c|c} A_0 & B \\ \hline B^T & A_1 \end{array}\right) \tag{24}$$

where

$$A_0 = \begin{pmatrix} I_{\lambda,\lambda} & I_{\lambda,\varepsilon} \\ I_{\lambda,\varepsilon} & I_{\varepsilon,\varepsilon} \end{pmatrix}, \quad B = \begin{pmatrix} I_{\lambda,\gamma} & I_{\lambda,p_1} & \ldots & I_{\lambda,p_n} \\ I_{\varepsilon,\gamma} & I_{\varepsilon,p_1} & \ldots & I_{\varepsilon,p_n} \end{pmatrix},$$

and

$$A_1 = \left(\begin{array}{c|ccc} 0 & 1 & \ldots & 1 \\ \hline 1 & I_{p_1,p_1} & \ldots & 0 \\ \vdots & \vdots & \ddots & \vdots \\ 1 & 0 & \ldots & I_{p_n,p_n} \end{array}\right).$$

The Fisher information can be straightforwardly inverted, by applying two blockwise inversion formulae. First, , the matrix A_1 needs to be inverted by blockwise inversion. Then the blockwise inversion formula that uses the inverse A_1^{-1} can be applied, which

results only in the inversion of 2×2 matrices. Due to the complexity of the resulting formulae, this is omitted here. Once the Fisher information is inverted, the row and columns corresponding to the nuisance parameter γ can be disregarded. Let the resulting matrix be denoted by $\mathcal{I}^{-1}$.

The average MOI ψ might be of more interest than the MOI parameter λ. Following the steps in [1] with obvious modifications yields the inverse Fisher information for the parameter vector $(\psi, \varepsilon, \boldsymbol{p})$, as

$$\tilde{\mathcal{I}}^{-1} = \begin{pmatrix} A^2 v_{\lambda,\lambda} & A v_{\lambda,\varepsilon} & A v_{\lambda,p_1} & \dots & A v_{\lambda,p_n} \\ A v_{\lambda,\varepsilon} & v_{\varepsilon,\varepsilon} & v_{\varepsilon,p_1} & \dots & v_{\varepsilon,p_n} \\ A v_{\lambda,p_1} & v_{\varepsilon,p_1} & v_{p_1,p_1} & \dots & v_{p_1,p_n} \\ \vdots & \vdots & \vdots & \ddots & \vdots \\ A v_{\lambda,p_n} & v_{\varepsilon,p_n} & v_{p_n,p_n} & \dots & v_{p_n,p_n} \end{pmatrix}, \tag{25a}$$

where

$$A = e^{\lambda} \frac{e^{\lambda} - \lambda - 1}{(e^{\lambda} - 1)^2} \tag{25b}$$

and the entries $v_{i,j}$ denote the entries of the inverse Fisher information.

Incomplete-data model (IDM) as a natural exponential family

First, we derive (31). We introduced $f_0(\boldsymbol{x}) = \frac{1}{2^n}$ (30) as the base density, which is uniform on the support of $\boldsymbol{x}$. The probability of observing a non-empty record (7a) can be rewritten as

$$\begin{aligned} \tilde{Q}_{\boldsymbol{x}} =& \frac{1}{2^n} \operatorname{Exp}\Bigg\{ \sum_{k=1}^{n} x_k \log\left(\frac{(1-\varepsilon)(e^{\lambda p_k} - 1)}{\varepsilon(e^{\lambda p_k} - 1) + 1} \right) + \sum_{k=1}^{n} \log\Big(\varepsilon(e^{\lambda p_k} - 1) + 1 \Big) \\ & - \log(e^{\lambda} - 1) + n \log 2 \Bigg\}. \end{aligned} \tag{26}$$

Similarly, the probability of observing an empty record is rewritten as

$$\begin{aligned} \tilde{Q}_{\boldsymbol{0}} =& \frac{1}{2^n} \operatorname{Exp}\Bigg\{ \log\Bigg(-1 + \prod_{k=1}^{n} \Big(\varepsilon(e^{\lambda p_k} - 1) + 1 \Big) \Bigg) - \log(e^{\lambda} - 1) + n \log 2 \Bigg\} \\ =& \frac{1}{2^n} \operatorname{Exp}\Bigg\{ \log\Bigg(1 - \frac{1}{\prod_{k=1}^{n} \Big(\varepsilon(e^{\lambda p_k} - 1) + 1 \Big)} \Bigg) + \sum_{k=1}^{n} \log\Big(\varepsilon(e^{\lambda p_k} - 1) + 1 \Big) \\ & - \log(e^{\lambda} - 1) + n \log 2 \Bigg\}. \end{aligned} \tag{27}$$

For $k = 1, \dots, n$, let

$$\begin{aligned} g_k(\boldsymbol{\theta}) :=& \log\left(\frac{(1-\varepsilon)(e^{\lambda p_k} - 1)}{\varepsilon(e^{\lambda p_k} - 1) + 1} \right) = -\log\left(\frac{\varepsilon(e^{\lambda p_k} - 1) + 1}{(1-\varepsilon)(e^{\lambda p_k} - 1)} \right) \\ =& -\log\left(\frac{e^{\lambda p_k}}{(1-\varepsilon)(e^{\lambda p_k} - 1)} - 1 \right) \\ =& -\log\left(\frac{1}{(1-\varepsilon)(1 - e^{-\lambda p_k})} - 1 \right), \end{aligned} \tag{28}$$

and additionally let

$$\begin{aligned} g_0(\boldsymbol{\theta}) &:= \log\left(1 - \frac{1}{\prod_{k=1}^{n}\Big(\varepsilon(e^{\lambda p_k}-1)+1\Big)}\right) \\ &= \log\left(1 - \frac{1}{\prod_{k=1}^{n}\Big(e^{\lambda p_k}\Big(\varepsilon(1-e^{-\lambda p_k})+e^{-\lambda p_k}\Big)\Big)}\right) \\ &= \log\left(1 - \frac{1}{e^{\lambda}\prod_{k=1}^{n}\Big(1-(1-e^{-\lambda p_k})+\varepsilon(1-e^{-\lambda p_k})\Big)}\right) \\ &= \log\left(1 - \frac{e^{-\lambda}}{\prod_{k=1}^{n}\Big(1-(1-\varepsilon)(1-e^{-\lambda p_k})\Big)}\right), \end{aligned} \tag{29}$$

and

$$\mathcal{G}(\boldsymbol{\theta}) := -\sum_{k=1}^{n}\log\Big(\varepsilon(e^{\lambda p_k}-1)+1\Big) + \log(e^{\lambda}-1) - n\log 2. \tag{30}$$

Thus, (26) and (27) can be rewritten as

$$\tilde{Q}_{\boldsymbol{x}} = \frac{1}{2^n}\operatorname{Exp}\left\{\sum_{k=1}^{n} x_k g_k(\boldsymbol{\theta}) - \mathcal{G}(\boldsymbol{\theta})\right\}, \tag{31a}$$

and

$$\tilde{Q}_{\boldsymbol{0}} = \frac{1}{2^n}\operatorname{Exp}\Big\{g_0(\boldsymbol{\theta}) - \mathcal{G}(\boldsymbol{\theta})\Big\}. \tag{31b}$$

Remember, for an empty record

$$\boldsymbol{h}(\boldsymbol{0}) = \Big(h_0(\boldsymbol{x}), h_1(\boldsymbol{x}), \ldots, h_n(\boldsymbol{x})\Big) = (1, 0, \ldots, 0),$$

and for a non-empty record

$$\boldsymbol{h}(\boldsymbol{x}) = \Big(h_0(\boldsymbol{x}), h_1(\boldsymbol{x}), \ldots, h_n(\boldsymbol{x})\Big) = (0, x_1, \ldots, x_n)$$

(cf. eq. 26). By using these facts we see that

$$\sum_{k=0}^{n}\boldsymbol{h}_k(\boldsymbol{x})g_k(\boldsymbol{\theta}) = \begin{cases} \sum_{k=1}^{n} x_k g_k(\boldsymbol{\theta}) & \text{if } \boldsymbol{x} \neq \boldsymbol{0}, \\ g_0(\boldsymbol{\theta}) & \text{if } \boldsymbol{x} = \boldsymbol{0}. \end{cases} \tag{32}$$

Therefore, (31) can be written jointly as

$$\tilde{Q}_{\boldsymbol{x}} = \operatorname{Exp}\left\{\sum_{k=0}^{n}\boldsymbol{h}_k(\boldsymbol{x})g_k(\boldsymbol{\theta}) - \mathcal{G}(\boldsymbol{\theta})\right\} f_0(\boldsymbol{x}) \tag{33}$$

for all $\boldsymbol{x} \in \tilde{\mathcal{O}}$.

To rewrite (31) into the natural form, we need to show that the function $\boldsymbol{g}$ defined by $\boldsymbol{\beta} = \boldsymbol{g}(\boldsymbol{\theta})$ (i.e., $\beta_k = g_k(\boldsymbol{\theta})$ for $k = 0, 1, \ldots, n$) is a 1-1 map between $\Theta = \mathbb{R}^+ \times \mathcal{S}_{n-1} \times (0,1)$ and $\Omega = \mathbb{R}^- \times \mathbb{R}^n$. Let $\boldsymbol{\beta} \in \Omega$ be arbitrarily chosen. From (28) and (29), we have

$$\beta_k = -\log\left(\frac{1}{(1-\varepsilon)(1-e^{-\lambda p_k})} - 1\right), \tag{34a}$$

and

$$\beta_0 = \log\left(1 - \frac{e^{-\lambda}}{\prod_{k=1}^{n}\left(1-(1-\varepsilon)(1-e^{-\lambda p_k})\right)}\right). \tag{34b}$$

By manipulating (34a) we obtain

$$(1-e^{-\lambda p_k})(1-\varepsilon) = \frac{1}{1+e^{-\beta_k}}, \tag{34c}$$

while substituting (34c) into (34b) gives

$$\beta_0 = \log\left(1 - \frac{e^{-\lambda}}{\prod_{k=1}^{n}\left(1 - \frac{1}{1+e^{-\beta_k}}\right)}\right) = \log\left(1 - e^{-\lambda}\prod_{k=1}^{n}\left(1+e^{\beta_k}\right)\right). \tag{34d}$$

Some algebraic manipulations yield

$$\lambda = -\log\left(1-e^{\beta_0}\right) + \sum_{k=1}^{n}\log\left(1+e^{\beta_k}\right). \tag{35}$$

Hence, λ is unique for any choice of $\boldsymbol{\beta}$. Additionally, from (34c) one derives

$$p_k = -\frac{1}{\lambda}\log\left(1 - \frac{1}{(1-\varepsilon)(1+e^{-\beta_k})}\right) \tag{36}$$

for all $k = 1, \ldots, n$. Since $\sum_{k=1}^{n} p_k = 1$ and λ is given by (35), the relation

$$1 = \frac{-\sum_{k=1}^{n}\log\left(1 - \frac{1}{(1-\varepsilon)(1+e^{-\beta_k})}\right)}{-\log\left(1-e^{\beta_0}\right) + \sum_{k=1}^{n}\log\left(1+e^{\beta_k}\right)} \tag{37}$$

holds. It only remains to show that this equation has exactly one solution for ε. It can be rewritten as

$$\frac{-\log\left(1-e^{\beta_0}\right) + \sum_{k=1}^{n}\log\left(\frac{1-\varepsilon(1+e^{\beta_k})}{1-\varepsilon}\right)}{-\log\left(1-e^{\beta_0}\right) + \sum_{k=1}^{n}\log\left(1+e^{\beta_k}\right)} = 0. \tag{38}$$

Let

$$f(\varepsilon) = \frac{-\log\left(1-e^{\beta_0}\right) + \sum_{k=1}^{n}\log\left(\frac{1-\varepsilon(1+e^{\beta_k})}{1-\varepsilon}\right)}{-\log\left(1-e^{\beta_0}\right) + \sum_{k=1}^{n}\log\left(1+e^{\beta_k}\right)} = 0. \tag{39}$$

Note that the denominator of $f(\varepsilon)$ is always positive for any $\boldsymbol{\beta} \in \mathbb{R}^- \times \mathbb{R}^n$. We have

$$f(0) = \frac{-\log\left(1 - e^{\beta_0}\right)}{-\log\left(1 - e^{\beta_0}\right) + \sum_{k=1}^{n} \log\left(1 + e^{\beta_k}\right)} > 0, \tag{40}$$

since $-\log(1 - e^{\beta_0}) > 0$ for $\beta_0 \in \mathbb{R}^-$. Furthermore, (39) implies that ε must satisfy $\frac{1 - \varepsilon(1 + e^{\beta_k})}{1 - \varepsilon} > 0$ or equivalently $\varepsilon < \frac{1}{1 + e^{\beta_k}}$ for all $k = 1, \ldots, n$. Consequently, we have $f(\varepsilon) \to -\infty$ as $\varepsilon \to \min_k \left(\frac{1}{1 + e^{\beta_k}}\right)$. The function $f(\varepsilon)$ is strictly decreasing in the open interval $\left(0, \min_k \left(\frac{1}{1 + e^{\beta_k}}\right)\right)$, because

$$f'(\varepsilon) = \frac{-\frac{1}{1-\varepsilon} \sum_{k=1}^{n} \frac{e^{\beta_k}}{1 - \varepsilon(1 + e^{\beta_k})}}{-\log\left(1 - e^{\beta_0}\right) + \sum_{k=1}^{n} \log\left(1 + e^{\beta_k}\right)} < 0. \tag{41}$$

Further, it can be easily checked that $f''(\varepsilon) < 0$, which means that $f(\varepsilon)$ is in addition strictly concave. Therefore, $f(\varepsilon) = 0$ has a unique solution in $\left(0, \min_k \left(\frac{1}{1 + e^{\beta_k}}\right)\right)$. The equation (39) can be solved iteratively for ε by a 1-dimensional Newton method. In detail, for an initial value $\varepsilon_0 \lesssim \min_k \left(\frac{1}{1 + e^{\beta_k}}\right)$, the iteration

$$\varepsilon_{t+1} = \varepsilon_t - \frac{-\log\left(1 - e^{\beta_0}\right) + \sum_{k=1}^{n} \log\left(\frac{1 - \varepsilon_t(1 + e^{\beta_k})}{1 - \varepsilon_t}\right)}{-\frac{1}{1-\varepsilon_t} \sum_{k=1}^{n} \frac{e^{\beta_k}}{1 - \varepsilon_t(1 + e^{\beta_k})}} \tag{42}$$

will converge to the desired solution. After, the solution is substituted into (36) the unique lineage frequencies can be derived.

Finally, we derive $\mathcal{B}(\boldsymbol{\beta})$. Note that $\mathcal{B}(\boldsymbol{\beta}) = \mathcal{G}(\boldsymbol{g}^{-1}(\boldsymbol{\beta}))$. However, the inverse of $\boldsymbol{g}$ has no closed form. This is irrelevant as long as $\mathcal{B}(\boldsymbol{\beta})$ can be derived explicitly.

First, note

$$\begin{aligned}
\mathcal{G}(\boldsymbol{\theta}) &= \log\left(\frac{e^{\lambda} - 1}{\prod_{k=1}^{n}\left(\varepsilon(e^{\lambda p_k} - 1) + 1\right)}\right) - n \log 2 \\
&= \log\left(\frac{1}{\prod_{k=1}^{n}\left(1 - (1-\varepsilon)(1 - e^{-\lambda p_k})\right)} - \frac{e^{-\lambda}}{\prod_{k=1}^{n}\left(1 - (1-\varepsilon)(1 - e^{-\lambda p_k})\right)}\right) - n \log 2.
\end{aligned}$$

From (34c) and (34b) we derive

$$\frac{1}{\prod_{k=1}^{n}\left(1 - (1-\varepsilon)(1 - e^{-\lambda p_k})\right)} = \prod_{k=1}^{n}\left(1 + e^{\beta_k}\right), \tag{43}$$

and

$$\frac{e^{-\lambda}}{\prod_{k=1}^{n}\left(1 - (1-\varepsilon)(1 - e^{-\lambda p_k})\right)} = 1 - e^{\beta_0}. \tag{44}$$

By substituting these terms in the above formula for $\mathcal{G}(\boldsymbol{\theta})$, one arrives at the desired form of $\mathcal{B}(\boldsymbol{\beta})$ (cf. eq. 35).

Minimality of the natural exponential family

To show that the natural exponential family (33) is minimal, we prove that there is no affine dependency between the components of $\boldsymbol{z}$, as well as between the components of $\boldsymbol{\beta}$. This is equivalent to showing that the sets $\{1, z_0(\boldsymbol{x}), z_1(\boldsymbol{x}), \ldots, z_n(\boldsymbol{x})\}$ and $\{1, \beta_0(\boldsymbol{\theta}), \beta_1(\boldsymbol{\theta}), \ldots, \beta_n(\boldsymbol{\theta})\}$ are linearly independent. To verify the former, we show that at least one of the generalized Wronskians of $\{1, z_0(\boldsymbol{x}), z_1(\boldsymbol{x}), \ldots, z_n(\boldsymbol{x})\}$ is non-zero [2,3], namely from (34) and (26) we obtain

$$\begin{vmatrix} 1 & z_1 & \cdots & z_n & z_0 \\ \frac{\partial 1}{\partial x_1} & \frac{\partial z_1}{\partial x_1} & \cdots & \frac{\partial z_n}{\partial x_1} & \frac{\partial z_0}{\partial x_1} \\ \vdots & \vdots & \ddots & \vdots & \vdots \\ \frac{\partial 1}{\partial x_n} & \frac{\partial z_1}{\partial x_n} & \cdots & \frac{\partial z_n}{\partial x_n} & \frac{\partial z_0}{\partial x_n} \\ \frac{\partial 1}{\partial x_1 \partial x_2} & \frac{\partial z_1}{\partial x_1 \partial x_2} & \cdots & \frac{\partial z_n}{\partial x_1 \partial x_2} & \frac{\partial z_0}{\partial x_1 \partial x_2} \end{vmatrix} = \begin{vmatrix} 1 & x_1 & \cdots & x_n & \prod_{k=1}^{n}(1-x_k) \\ 0 & 1 & \cdots & 0 & -\prod_{k=2}^{n}(1-x_k) \\ \vdots & \vdots & \ddots & \vdots & \vdots \\ 0 & 0 & \cdots & 1 & -\prod_{k=1}^{n-1}(1-x_k) \\ 0 & 0 & \cdots & 0 & \prod_{k=3}^{n}(1-x_k) \end{vmatrix}$$

$$= \prod_{k=3}^{n}(1-x_k) \not\equiv 0.$$

In order to show that the set $\{1, \beta_0(\boldsymbol{\theta}), \beta_1(\boldsymbol{\theta}), \ldots, \beta_n(\boldsymbol{\theta})\}$ is linearly independent, we introduce the transformations

$$\eta_0 = 1 - \varepsilon \tag{45a}$$

and

$$\eta_k = e^{\lambda p_k} \tag{45b}$$

for $k = 1, \ldots, n$. This transformation defines a 1-1 map $T : \Theta \to (0,1) \times (1, +\infty)^n$, by

$$T(\boldsymbol{\theta}) = \left(1 - \varepsilon, e^{\lambda p_1}, \ldots, e^{\lambda p_n}\right) = \left(\eta_0, \eta_1, \ldots, \eta_n\right) =: \boldsymbol{\eta}, \tag{46}$$

where the inverse of T is given by

$$T^{-1}(\eta) = \left(1 - \eta_0, \sum_{k=1}^{n} \log \eta_k, \frac{\log \eta_1}{\sum_{k=1}^{n} \log \eta_k}, \ldots, \frac{\log \eta_n}{\sum_{k=1}^{n} \log \eta_k}\right). \tag{47}$$

By this transformation, we eliminate one of the redundant parameters, namely, one of the lineage frequencies since they vary on the $(n-1)$-dimensional simplex. Now, it suffices to show that the Jacobian of $\boldsymbol{\beta}(\boldsymbol{\eta})$ has full rank. Some algebraic manipulations yield that (34) can be written in terms of the new parameters as

$$\beta_0(\boldsymbol{\eta}) = \log\left(1 - \frac{1}{\prod_{l=1}^{n}\left(\eta_l - \eta_0(1 - \eta_l)\right)}\right) \tag{48}$$

and

$$\beta_k(\boldsymbol{\eta}) = -\log\left(\frac{\eta_k}{\eta_0(\eta_k - 1)} - 1\right). \tag{49}$$

The Jacobian of $\boldsymbol{\beta}(\boldsymbol{\eta})$ is

$$\mathbb{J}_{\boldsymbol{\beta}} = \begin{pmatrix} \frac{\partial\beta_0}{\partial\eta_0} & \frac{\partial\beta_0}{\partial\eta_1} & \cdots & \frac{\partial\beta_0}{\partial\eta_n} \\ \frac{\partial\beta_1}{\partial\eta_0} & \frac{\partial\beta_1}{\partial\eta_1} & \cdots & \frac{\partial\beta_1}{\partial\eta_n} \\ \vdots & \vdots & \ddots & \vdots \\ \frac{\partial\beta_n}{\partial\eta_0} & \frac{\partial\beta_n}{\partial\eta_1} & \cdots & \frac{\partial\beta_n}{\partial\eta_n} \end{pmatrix}. \tag{50}$$

We need the following partial derivatives

$$\frac{\partial\beta_0}{\partial\eta_0} = -\frac{1}{-1 + \prod_{l=1}^{n}\Big(\eta_l - \eta_0(1-\eta_l)\Big)} \sum_{l=1}^{n} \frac{\eta_l - 1}{\eta_l - \eta_0(\eta_l - 1)}, \tag{51a}$$

$$\frac{\partial\beta_0}{\partial\eta_k} = \frac{1}{-1 + \prod_{l=1}^{n}\Big(\eta_l - \eta_0(1-\eta_l)\Big)} \frac{1-\eta_0}{\eta_k - \eta_0(\eta_k - 1)}, \tag{51b}$$

$$\frac{\partial\beta_k}{\partial\eta_0} = \frac{\eta_k}{\eta_0} \frac{1}{\eta_k - \eta_0(\eta_k - 1)}, \tag{51c}$$

$$\frac{\partial\beta_k}{\partial\eta_k} = \frac{1}{\eta_k - 1} \frac{1}{\eta_k - \eta_0(\eta_k - 1)}, \tag{51d}$$

and

$$\frac{\partial\beta_k}{\partial\eta_l} = 0, \tag{51e}$$

where the last term is true for $l \neq k$.

To determine the rank of $\mathbb{J}_{\boldsymbol{\beta}}$ we apply a series of elementary row operations to reduce $\mathbb{J}_{\boldsymbol{\beta}}$ to a matrix in row echelon form. Namely, we multiply the $(k+1)$-th row with the function

$$-\frac{(\eta_k - 1)(1-\eta_0)}{-1 + \prod_{l=1}^{n}\Big(\eta_l - \eta_0(\eta_l - 1\Big)}, \tag{52}$$

and add it to the first row for all $k = 1, \ldots, n$. This yields

$$\begin{pmatrix} a & 0 & \cdots & 0 \\ \frac{\eta_1}{\eta_0}\frac{1}{\eta_1 - \eta_0(\eta_1 - 1)} & \frac{1}{\eta_1 - 1}\frac{1}{\eta_1 - \eta_0(\eta_1 - 1)} & \cdots & 0 \\ \vdots & \vdots & \ddots & \vdots \\ \frac{\eta_n}{\eta_0}\frac{1}{\eta_n - \eta_0(\eta_n - 1)} & 0 & \cdots & \frac{1}{\eta_n - 1}\frac{1}{\eta_n - \eta_0(\eta_n - 1)} \end{pmatrix}, \tag{53}$$

where

$$a = \frac{1}{-1 + \prod_{l=1}^{n}\Big(\eta_l - \eta_0(1-\eta_l)\Big)} \left(\frac{\eta_0 - 1}{\eta_0} \sum_{l=1}^{n} \frac{\eta_l(\eta_l - 1)}{\eta_l - \eta_0(\eta_l - 1)} - \sum_{l=1}^{n} \frac{\eta_l - 1}{\eta_l - \eta_0(\eta_l - 1)}\right). \tag{54}$$

Clearly, the matrix (53) is in echelon form and has full rank.

Interpretation of the MLE

Here, we present the proof of Result 1, which gives an intuitive interpretation of the MLE.
Proof of Result 1. The assertion can be proved without explicitly calculating the MLE. Implicitly, the MLE is given by (48) in terms of the parameters of the natural exponential family, $\boldsymbol{\beta}$.

Let

$$E := \prod_{k=1}^{n}\Big(1-(1-\varepsilon)(1-e^{-\lambda p_k})\Big). \tag{55}$$

Rearrangement of (7c) yields

$$\begin{aligned}\hat{\hat{Q}}_{\mathbf{0}} &= \frac{1}{e^{\hat{\lambda}}-1}\Bigg(-1+\prod_{k=1}^{n} e^{\hat{\lambda}\hat{p}_k}\Big(\varepsilon(1-e^{-\hat{\lambda}\hat{p}_k})+e^{-\hat{\lambda}\hat{p}_k}\Big)\Bigg)\\ &= \frac{1}{e^{\hat{\lambda}}-1}\Bigg(-1+\prod_{k=1}^{n} e^{\hat{\lambda}\hat{p}_k}\prod_{k=1}^{n}\Big(\varepsilon(1-e^{-\hat{\lambda}\hat{p}_k})+e^{-\hat{\lambda}\hat{p}_k}\Big)\Bigg)\\ &= \frac{e^{\hat{\lambda}}}{e^{\hat{\lambda}}-1}\Bigg(-e^{-\hat{\lambda}}+\prod_{k=1}^{n}\Big(\varepsilon(1-e^{-\hat{\lambda}\hat{p}_k})+e^{-\hat{\lambda}\hat{p}_k}\Big)\Bigg)\\ &= \frac{e^{\hat{\lambda}}}{e^{\hat{\lambda}}-1}\Bigg(-e^{-\hat{\lambda}}+\prod_{k=1}^{n}\Big(1-(1-\varepsilon)(1-e^{-\lambda p_k})\Big)\Bigg),\end{aligned}$$

i.e.,

$$\hat{\hat{Q}}_{\mathbf{0}} = \frac{1}{1-e^{-\hat{\lambda}}}(E-e^{-\hat{\lambda}}). \tag{56}$$

Note that, (48) yields

$$\frac{n_{\mathbf{0}}}{N} = \frac{e^{\hat{\beta}_0}}{e^{\hat{\beta}_0}-1+D}, \tag{57a}$$

and

$$\frac{N_k}{N} = \frac{e^{\hat{\beta}_k}}{1+e^{\hat{\beta}_k}}\frac{D}{e^{\hat{\beta}_0}-1+D}, \tag{57b}$$

where

$$D := \prod_{k=1}^{n}(1+e^{\hat{\beta}_k}) = \frac{1}{E}, \tag{58}$$

by using (43). Moreover, (44) yields

$$e^{\hat{\beta}_0}-1 = -\frac{e^{-\hat{\lambda}}}{E} \quad \text{or} \quad e^{\hat{\beta}_0} = \frac{E-e^{-\hat{\lambda}}}{E}. \tag{59}$$

Therefore,(57b) can be rewritten as

$$\frac{N_k}{N} = \frac{e^{\hat{\beta}_k}}{1+e^{\hat{\beta}_k}}\frac{\frac{1}{E}}{\frac{1}{E}-\frac{e^{-\hat{\lambda}}}{E}} = \frac{e^{\hat{\beta}_k}}{1+e^{\hat{\beta}_k}}\frac{1}{1-e^{-\hat{\lambda}}},$$

which by (34c), (9) and (10) yields

$$\frac{N_k}{N} = (1 - \hat{\varepsilon})\hat{q}_k = \hat{\hat{q}}_k, \tag{60}$$

which is the third assertion. Furthermore, by using (58) and (59) one can rewrite (57a) as

$$\frac{n_{\mathbf{0}}}{N} = \frac{E - e^{-\hat{\lambda}}}{1 - e^{-\hat{\lambda}}}, \tag{61}$$

which yields

$$E = \frac{n_{\mathbf{0}}}{N}(1 - e^{-\hat{\lambda}}) + e^{-\hat{\lambda}}. \tag{62}$$

Thus, (56) is derived as

$$\hat{\hat{Q}}_{\mathbf{0}} = \frac{1}{1 - e^{-\hat{\lambda}}}\left(e^{-\hat{\lambda}} + (1 - e^{-\hat{\lambda}})\frac{n_{\mathbf{0}}}{N} - e^{-\hat{\lambda}}\right) = \frac{n_{\mathbf{0}}}{N},$$

proving the first statement. Finally, by using (9) and (55), (11) can be rewritten as

$$\hat{\hat{q}}_k^{(c)} = (1 - \hat{\varepsilon})\hat{q}_k \frac{1 - e^{-\hat{\lambda}}}{1 - E}. \tag{63}$$

By substituting (61) into the above and using (60) one obtains

$$\hat{\hat{q}}_k^{(c)} = (1 - \hat{\varepsilon})\hat{q}_k \frac{N}{N - n_{\mathbf{0}}} = \frac{N_k}{N - n_{\mathbf{0}}} = \frac{N_k}{N_+},$$

which proves the second statement.

□

Pathological cases

Here, we switch between the original parametrization $\boldsymbol{\theta} = (\lambda, \boldsymbol{p}, \varepsilon)$ and the natural parameters of the exponential family $\boldsymbol{\beta}$. The following calculations need to be understood in a limiting sense. We distinguish between three cases: (A) $n_{\mathbf{0}} = 0$, (B) $0 < \frac{n_{\mathbf{0}}}{N} < \prod_{l=1}^{n}\left(1 - \frac{N_l}{N}\right)$, and (C) $0 < \prod_{l=1}^{n}\left(1 - \frac{N_l}{N}\right) \le \frac{n_{\mathbf{0}}}{N}$.

(A) Note, $n_{\mathbf{0}} = 0$ is equivalent to $N = N_+$. In this case $\boldsymbol{U} \notin \operatorname{int}\mathcal{C}(\mathcal{U})$, hence the MLE $\hat{\beta}$ lies on the boundary of the parameters space $\mathbb{R}^{n+1}$. Equivalently, $\boldsymbol{\theta}$ lies on the boundary of the parameter space Θ. The condition, $N = N_+$ implies $\hat{\beta}_0 = -\infty$ according to (48a) or equivalently $\hat{\varepsilon} = 0$. We distinguish four sub-cases.

(A.1) Assume $\sum_{k=1}^{n} N_k > N$ and $0 < N_k < N$ for all k. Because the likelihood function of the IDM for $\varepsilon = 0$ coincides with that of the OM, the results of [4] imply $\hat{\lambda} > 0$ and $\hat{p}_k > 0$ for all k.

(A.2) If $\sum_{k=1}^{n} N_k = N$ but $0 < N_k < N$ for all k, $\hat{\beta}_k = \infty$ is impossible because (48b) would imply $N_k = N$. Hence, $\hat{\beta}_k = -\infty$ for all k, and (48b) implies $\prod_{l=1}^{n}\left(1 + e^{\hat{\beta}_l}\right) = 1$ or $e^{\hat{\beta}_k} = 0$ for all k, since $e^{\hat{\beta}_0} = 0$. This translates into $\hat{\lambda} = 0$ since not all p_k can vanish. From this $\frac{e^{\hat{\beta}_k}(1 + e^{\hat{\beta}_l})}{e^{\hat{\beta}_l}(1 + e^{\hat{\beta}_k})} = \frac{N_k}{N_l}$ follows, which translates into $\frac{\hat{p}_k}{\hat{p}_l} = \frac{N_k}{N_l}$, or $\hat{p}_k = \frac{N_k}{N}$.

(A.3) Assume $N_k = N$ and $N_j > 0$ for at least one $j \neq k$. By equating $N_k = N$ in (48b), some algebraic manipulations yields $\prod_{l=1}^{n}\left(1+e^{\hat{\beta}_l}\right) = 1 + e^{\hat{\beta}_k}$. The latter implies $\hat{\beta}_k = \infty$, or $\hat{\lambda} = \infty$ because $\hat{\varepsilon} = 0$. (Note that $\hat{\beta}_l = -\infty$ for all $l \neq k$, is impossible because it would imply $N_l = 0$, a contradiction.) Furthermore, if for lineage A_j we have $N_j < N$, (48b) implies

$$\frac{N_j}{N} = \frac{e^{\hat{\beta}_j}}{1+e^{\hat{\beta}_j}} \frac{\prod_{l=1}^{n}\left(1+e^{\hat{\beta}_l}\right)}{-1+\prod_{l=1}^{n}\left(1+e^{\hat{\beta}_l}\right)} = \frac{e^{\hat{\beta}_j}}{1+e^{\hat{\beta}_j}} \frac{1+e^{\hat{\beta}_k}}{e^{\hat{\beta}_k}}. \tag{64}$$

Because $\hat{\beta}_k = \infty$, this yields

$$\frac{N_j}{N} = \frac{e^{\hat{\beta}_j}}{1+e^{\hat{\beta}_j}}. \tag{65}$$

In terms of the original parameters this translates to

$$\hat{\lambda}\hat{p}_j = -\log\left(1 - \frac{N_j}{N}\right),$$

which has to be understood in a limit sense, i.e., the likelihood function reaches a supremum in any limit $\lambda \to \infty$ such that $p_j \to 0$ but

$$\lambda p_j \to -\log\left(1 - \frac{N_j}{N}\right) \text{ if } N_j < N, \text{ and } \lambda p_k \to \infty \text{ if } N_k = N, \tag{66}$$

subject to the constraint $\sum_{l=1}^{n} \hat{p}_l = 1$. In particular, in the case that $N_k = N$ for two or more k, the supremum of the likelihood function is a continuum.

(A.4) If $N_k \neq 0$ for only one k, obviously the likelihood function attains its maximum for any $\hat{\lambda} \geq 0$ and $\hat{p}_k = 1$.

(B) Now assume $0 < \frac{n_{\mathbf{0}}}{N} < \prod_{l=1}^{n}\left(1 - \frac{N_l}{N}\right)$. Further assume $\sum_{k=1}^{n} N_k = N_+$, but $0 < N_k < N_+$ for all k. Also in this case $\boldsymbol{U} \notin \operatorname{int}\mathcal{C}(\mathcal{U})$ and the estimate lies on the boundary of the admissible parameter space. Notice that $\hat{\beta}_k = \infty$ for one or more k is impossible, because (48b) would then yield $N_k = N$, which would imply $n_{\mathbf{0}} = 0$. Hence, $\hat{\beta}_k = -\infty$ for all k which also implies $\hat{\beta}_0 = -\infty$ because by (48a) we have

$$e^{\hat{\beta}_0} = \frac{n_{\mathbf{0}}}{N_+}\left(-1 + \prod_{l=1}^{n}\left(1+e^{\hat{\beta}_l}\right)\right). \tag{67}$$

This translates into $\hat{\lambda} = 0$ since not all p_k can vanish. However, the MLE for ε is found in the limit $\lambda \to 0$. By replacing $\hat{\boldsymbol{\beta}}$ in (48a) with its equivalent in (28) in terms of the original parametrization, we derive that the estimate satisfies

$$\frac{n_{\mathbf{0}}}{N} = \frac{-e^{-\lambda} + \prod_{l=1}^{n}\Big(1-(1-\varepsilon)(1-e^{-\lambda p_l})\Big)}{1-e^{-\lambda}} \tag{68}$$

in the limit $\lambda \to 0$. The limit is derived by applying the l'Hospital's rule to the right -hand side of (68), i.e.,

$$\frac{n_{\mathbf{0}}}{N} = \lim_{\lambda\to 0} \frac{e^{-\lambda} - \prod_{l=1}^{n}\Big(1-(1-\varepsilon)(1-e^{-\lambda p_l})\Big)\sum_{l=1}^{n}\frac{(1-\varepsilon)p_l e^{-\lambda p_l}}{1-(1-\varepsilon)(1-e^{-\lambda p_l})}}{e^{-\lambda}} = \varepsilon. \tag{69}$$

Therefore, $\hat{\varepsilon} = \dfrac{n_{\mathbf{0}}}{N}$. Additionally, by using (67) and (48b), we derive

$$\frac{e^{\hat{\beta}_k}}{1+e^{\hat{\beta}_k}} \frac{\prod_{l=1}^{n}\left(1+e^{\hat{\beta}_l}\right)}{\dfrac{N}{N_+}\left(-1+\prod_{l=1}^{n}\left(1+e^{\hat{\beta}_l}\right)\right)} = \frac{N_k}{N}, \tag{70}$$

which again by replacing $\hat{\boldsymbol{\beta}}$ with its equivalent in (28) gives

$$\frac{N_k}{N_+} = \frac{(1-\varepsilon)(1-e^{-\lambda p_k})}{1-\prod_{l=1}^{n}\left(1-(1-\varepsilon)(1-e^{-\lambda p_l})\right)}. \tag{71}$$

Taking the limit $\lambda \to 0$ and applying l'Hospital's rule yields $\hat{p}_k = \dfrac{N_k}{N_+}$.

For the sake of completeness, if $n_{\mathbf{0}} = N$, (48a) yields $\prod_{l=1}^{n}\left(1+e^{\hat{\beta}_l}\right) = 1$ which implies $\hat{\beta}_k = -\infty$ for all k. This can be translated into $\hat{\lambda} = 0$ or $\hat{\varepsilon} = 1$. However, $\hat{\lambda} = 0$ yields $e^{\hat{\beta}_0} = 0$ which contradicts (48a). Therefore, $\hat{\varepsilon} = 1$ and $\hat{\lambda}$ and $\hat{p}_k$ are arbitrary.

(C) If $\boldsymbol{U} \in \operatorname{int}\mathcal{C}(\mathcal{U})$, $\hat{\boldsymbol{\beta}}$ lies in the interior of the natural parameter space $\mathbb{R}^{n+1}$, but not necessarily in Ω. In this case, the original parameter vector $\boldsymbol{\theta}$ lies outside the admissible parameter space Θ. This occurs if $\hat{\beta}_0 \geq 0$.

We will distinguish between $\hat{\beta}_0 = 0$ and $\hat{\beta}_0 > 0$, for which the former results in a limit case in terms of the original parameters. Notably, $\beta_0 = 0$ is not at the boundary of the natural parameter space $\mathbb{R}^{n+1}$, however, it maps into a boundary point of the original parameter space Θ. In other words, for $\beta_0 = 0$ and arbitrary $\beta_k \in \mathbb{R}$ for all k, there exists no $\boldsymbol{\theta} \in \Theta$ such that $\boldsymbol{g}(\boldsymbol{\theta}) = \boldsymbol{\beta}$. We show that this case occurs if $\prod_{l=1}^{n}\left(1-\dfrac{N_l}{N}\right) = \dfrac{n_{\mathbf{0}}}{N}$ for $0 < n_{\mathbf{0}} < N$. To facilitate the calculations, let $\Gamma = \prod_{l=1}^{n}\left(1+e^{\beta_l}\right)$. We recognize (67) yields

$$\Gamma = \frac{N_+}{n_{\mathbf{0}}} e^{\beta_0} + 1. \tag{72}$$

Substituting in (48b) gives

$$\frac{e^{\beta_k}}{1+e^{\beta_k}} \frac{1}{N}\left(n_{\mathbf{0}} e^{-\beta_0} + N_+\right) = \frac{N_k}{N}, \tag{73}$$

which implies

$$e^{\beta_k} = \frac{N_k}{n_{\mathbf{0}} e^{-\beta_0} + N_+ - N_k}. \tag{74}$$

Hence, we derive

$$\Gamma = \prod_{l=1}^{n}\left(1+e^{\beta_l}\right) = \prod_{l=1}^{n}\left(\frac{n_{\mathbf{0}} e^{-\beta_0} + N_+}{n_{\mathbf{0}} e^{-\beta_0} + N_+ - N_l}\right) = \frac{1}{\prod_{l=1}^{n}\left(1-\dfrac{N_l}{n_{\mathbf{0}} e^{-\beta_0} + N_+}\right)}. \tag{75}$$

If this equation is substituted into (48a), one obtains

$$\frac{n_{\mathbf{0}}}{N} = \frac{e^{\beta_0}}{e^{\beta_0} - 1 + \dfrac{1}{\prod_{l=1}^{n}\left(1-\dfrac{N_l}{n_{\mathbf{0}} e^{-\beta_0} + N_+}\right)}}. \tag{76}$$

For $\hat{\beta}_0 = 0$ one obtains

$$\frac{n_{\mathbf{0}}}{N} = \prod_{l=1}^{n}\Big(1 - \frac{N_l}{N}\Big). \tag{77}$$

Furthermore, for $\hat{\beta}_0 = 0$, (48b) yields

$$\frac{e^{\hat{\beta}_k}}{1 + e^{\hat{\beta}_k}} = \frac{N_k}{N}. \tag{78}$$

for any $k = 1, \dots, n$. In this case, $\hat{\beta}_k = -\infty$ is impossible, as it would result in $N_k = 0$. Moreover, $\hat{\beta}_k = \infty$ is also impossible since it would yield $N_k = N$ or equivalently $n_{\mathbf{0}} = 0$. Notice that $\hat{\beta}_0 = 0$ translates into $\hat{\lambda} = \infty$. Formally, replacing β_k in (78), with the expression in (34c) implies that

$$\hat{\lambda}\hat{p}_k = -\log\Big(1 - \frac{N_k}{N}\frac{1}{1-\hat{\varepsilon}}\Big), \tag{79}$$

must hold for all k. Because $\hat{\lambda} = \infty$, this holds only if $\hat{\varepsilon} = 1 - \frac{N_k}{N}$ or $\hat{p}_k = 0$, and the constraint $\sum_{l=1}^{n} p_l = 1$ implies that $\hat{\lambda}\hat{p}_k = \infty$ for some k. Since $\hat{\lambda} = \infty$, no maximum is attained and a supremum is found in the limit $\lambda \to \infty$. In terms of original parameters, if $\hat{\varepsilon} = 1 - \frac{N_k}{N}$, (79) implies that the supremum is reached in any limit $\lambda \to \infty$ such that $p_j \to 0$ but

$$\lambda p_j \to -\log\left(1 - \frac{N_j}{N_k}\right) \text{ if } 1 - \frac{N_j}{N} > \hat{\varepsilon} = 1 - \frac{N_k}{N}.$$

Since $\lambda > 0$ and $p_j \geq 0$, the above relations imply $N_k = \max_j(N_j)$ or $\hat{\varepsilon} = \min_j\Big(1 - \frac{N_j}{N}\Big)$.

In summary, the supremum is attained in any limit $\lambda \to \infty$ such that

$$\varepsilon = \min_j\left(1 - \frac{N_j}{N}\right), \tag{80a}$$

$$\lambda p_k \to \begin{cases} -\log\left(1 - \dfrac{N_k}{\max_j(N_j)}\right) & \text{if } N_k < \max_j(N_j), \\ \infty & \text{if } \ N_k = \max_j(N_j), \end{cases} \tag{80b}$$

subject to the constraint $\sum_{l=1}^{n} p_l = 1$.

The case $\hat{\beta}_0 = 0$ holds hence under the condition $\frac{n_{\mathbf{0}}}{N} = \prod_{l=1}^{n}\Big(1 - \frac{N_k}{N}\Big)$, in which case the supremum (80) is reached.

Additionally, we shall note the following. For $\hat{\beta}_0 = 0$, $\frac{N_k}{N} = 1$ is impossible because $n_{\mathbf{0}} > 0$. If $N_k = N_+$ (i.e., $\frac{n_{\mathbf{0}} + N_k}{N} = 1$) the expression for $n_{\mathbf{0}}/N$ implies $N_j = 0$ for all $j \neq k$. Hence, $\hat{\lambda} = \infty$, $\hat{p}_k = 1$ and $\hat{\varepsilon} = \frac{n_{\mathbf{0}}}{N}$. This case is already subsumed by (80).

Finally, we show that (77) yields $\hat{\beta}_0 > 0$ if $\frac{n_{\mathbf{0}}}{N} > \prod_{l=1}^{n}\left(1 - \frac{N_k}{N}\right)$. Let us assume $\hat{\beta}_0 < 0$. We prove that this yields a contradiction. We have

$$\prod_{l=1}^{n}\left(1 - \frac{N_k}{N}\right) > \prod_{l=1}^{n}\left(1 - \frac{N_k}{n_{\mathbf{0}}e^{-\beta_0} + N_+}\right). \tag{81}$$

This inequality in combination with (76) gives

$$\frac{e^{\beta_0}}{e^{\beta_0} - 1 + \frac{1}{\prod_{l=1}^{n}\left(1 - \frac{N_l}{N}\right)}} > \frac{n_{\mathbf{0}}}{N}. \tag{82}$$

This can be rearranged as

$$e^{\beta_0}\left(1 - \frac{n_{\mathbf{0}}}{N}\right)\prod_{l=1}^{n}\left(1 - \frac{N_k}{N}\right) > \frac{n_{\mathbf{0}}}{N}\left(1 - \prod_{l=1}^{n}\left(1 - \frac{N_k}{N}\right)\right). \tag{83}$$

Since we assumed $\beta_0 < 0$, we can conclude

$$\left(1 - \frac{n_{\mathbf{0}}}{N}\right)\prod_{l=1}^{n}\left(1 - \frac{N_k}{N}\right) > \frac{n_{\mathbf{0}}}{N}\left(1 - \prod_{l=1}^{n}\left(1 - \frac{N_k}{N}\right)\right), \tag{84}$$

which after rearrangement yields

$$\prod_{l=1}^{n}\left(1 - \frac{N_k}{N}\right) > \frac{n_{\mathbf{0}}}{N}, \tag{85}$$

contradicting the assumption. Therefore, $\frac{n_{\mathbf{0}}}{N} > \prod_{l=1}^{n}\left(1 - \frac{N_k}{N}\right)$ results in $\hat{\beta}_0 > 0$, for which we cannot find a solution in the original parameter space Θ. Hence, the natural parameter space needs to be restricted to $\beta_0 \leq 0$. Since no maximum is attained in $\mathbb{R}^- \times \mathbb{R}^n$, a boundary maximum must be attained at $\beta_0 = 0$, which yields exactly the limit case (80b). □

The EM Algorithm

The EM algorithm finds the maximum-likelihood estimates of the model parameters $\boldsymbol{\theta}$ by a two-step iterative procedure. Given an initial value $\boldsymbol{\theta}^{(0)}$, the algorithm generates a sequence $\boldsymbol{\theta}^{(t)}$ until it converges to the MLE $\hat{\boldsymbol{\theta}}$. The convergence is imposed by requiring that, e.g., the Euclidean distance, difference of consecutive values of the sequence, i.e., $||\boldsymbol{\theta}^{(t+1)} - \boldsymbol{\theta}^{(t)}||_2$ is smaller than some small but arbitrary threshold δ, e.g., $\delta = 10^{-8}$. As before, let $\mathcal{X}$ be a dataset of size N containing observation vectors $\boldsymbol{x}^{(1)}, \ldots, \boldsymbol{x}^{(N)}$, which can be regarded as observed information corresponding to the unobserved MOI configurations $\boldsymbol{m}^{(1)}, \ldots, \boldsymbol{m}^{(N)}$. For each j we have $\boldsymbol{x}^{(j)} = (x_1^{(j)}, \ldots, x_n^{(j)})$ and $\boldsymbol{m}^{(j)} = (m_1^{(j)}, \ldots, m_n^{(j)})$. Let $\mathcal{M}$ denote the unobserved dataset corresponding to $\mathcal{X}$. In each iteration, the current parameter choice is $\boldsymbol{\theta}^{(t)} = (\lambda^{(t)}, \boldsymbol{p}^{(t)}, \varepsilon^{(t)})$. First, the expectation step is carried out followed by the maximization step.

Expectation step

In step t, first we calculate the conditional expectation of the Q-function which is the expectation of the log-likelihood function of the model parameters $\boldsymbol{\theta}$ given the

unobservable $\mathcal{M}$ (and hence the observable data $\mathcal{X}$) with respect to the conditional probability of $\mathcal{M}$ conditioned on the observable data $\mathcal{X}$ and the current parameter choice $\boldsymbol{\theta}^{(t)}$. In mathematical terms, the Q-function is defined as

$$Q\big(\boldsymbol{\theta}\,|\,\boldsymbol{\theta}^{(t)}\big) \coloneqq \mathbb{E}_{\mathcal{M}|\mathcal{X},\boldsymbol{\theta}^{(t)}}\Big(\log P\big(\mathcal{M},\mathcal{X}\,|\,\boldsymbol{\theta}\big)\Big). \tag{86}$$

Note that the parameter choice in step t in the Q-function, $\boldsymbol{\theta}^{(t)}$, is fixed, while the parameters $\boldsymbol{\theta}$ are considered variables. We can also write the Q-function as follows

$$Q\big(\boldsymbol{\theta}\,|\,\boldsymbol{\theta}^{(t)}\big) = \mathbb{E}_{\boldsymbol{m}^{(1)},\ldots,\boldsymbol{m}^{(N)}\,|\,\boldsymbol{x}^{(1)},\ldots,\boldsymbol{x}^{(N)},\boldsymbol{\theta}^{(t)}}\left(\log P\Big(\boldsymbol{m}^{(1)},\ldots,\boldsymbol{m}^{(N)},\boldsymbol{x}^{(1)},\ldots,\boldsymbol{x}^{(N)}\,\big|\,\boldsymbol{\theta}\Big)\right).$$

Since the infections are independent and have the same probability mass function, the above leads to

$$\begin{aligned}Q\big(\boldsymbol{\theta}\,|\,\boldsymbol{\theta}^{(t)}\big) &= \mathbb{E}_{\boldsymbol{m}^{(1)},\ldots,\boldsymbol{m}^{(N)}\,|\,\boldsymbol{x}^{(1)}\ldots,\boldsymbol{x}^{(N)},\boldsymbol{\theta}^{(t)}}\left(\log\left(\prod_{j=1}^{N}P\Big(\boldsymbol{m}^{(j)},\boldsymbol{x}^{(j)}\,\big|\,\boldsymbol{\theta}\Big)\right)\right)\\ &= \sum_{j=1}^{N}\mathbb{E}_{\boldsymbol{m}^{(1)},\ldots,\boldsymbol{m}^{(N)}\,|\,\boldsymbol{x}^{(1)}\ldots,\boldsymbol{x}^{(N)},\boldsymbol{\theta}^{(t)}}\left(\log P\Big(\boldsymbol{m}^{(j)},\boldsymbol{x}^{(j)}\,\big|\,\boldsymbol{\theta}\Big)\right).\end{aligned}$$

From marginalization, it is easily seen that

$$\begin{aligned}&\mathbb{E}_{\boldsymbol{m}^{(1)},\ldots,\boldsymbol{m}^{(N)}\,|\,\boldsymbol{x}^{(1)}\ldots,\boldsymbol{x}^{(N)},\boldsymbol{\theta}^{(t)}}\left(\log P\Big(\boldsymbol{m}^{(j)},\boldsymbol{x}^{(j)}\,\big|\,\boldsymbol{\theta}\Big)\right)\\ &= \mathbb{E}_{\boldsymbol{m}^{(j)}\,|\,\boldsymbol{x}^{(j)},\boldsymbol{\theta}^{(t)}}\left(\log P\Big(\boldsymbol{m}^{(j)},\boldsymbol{x}^{(j)}\,\big|\,\boldsymbol{\theta}\Big)\right).\end{aligned}$$

Hence, the Q-function becomes

$$Q\big(\boldsymbol{\theta}\,|\,\boldsymbol{\theta}^{(t)}\big) = \sum_{j=1}^{N}\mathbb{E}_{\boldsymbol{m}^{(j)}\,|\,\boldsymbol{x}^{(j)},\boldsymbol{\theta}^{(t)}}\left(\log P\Big(\boldsymbol{m}^{(j)},\boldsymbol{x}^{(j)}\,\big|\,\boldsymbol{\theta}\Big)\right).$$

Deriving the Q-function is lengthy. Let

$$Q_j\big(\boldsymbol{\theta}\,|\,\boldsymbol{\theta}^{(t)}\big) \coloneqq \mathbb{E}_{\boldsymbol{m}^{(j)}\,|\,\boldsymbol{x}^{(j)},\boldsymbol{\theta}^{(t)}}\left(\log P\Big(\boldsymbol{m}^{(j)},\boldsymbol{x}^{(j)}\,\big|\,\boldsymbol{\theta}\Big)\right), \tag{87}$$

and $\boldsymbol{x}^{(j)} \neq \mathbf{0}$. Additionally, let $m^{(j)} = \sum\limits_{k=1}^{n} m_k^{(j)}$ be the MOI of sample j, and $\boldsymbol{y}^{(j)} \coloneqq \operatorname{sign}\big(\boldsymbol{m}^{(j)}\big)$, i.e., $y_k^{(j)} = \operatorname{sign}\big(m_k^{(j)}\big)$ for $k = 1,\ldots,n$. Hence, $\boldsymbol{y}^{(j)}$ indicates the actual absence/presence of lineages in the j-th infection. Clearly $\boldsymbol{x}^{(j)} \leq \boldsymbol{y}^{(j)}$. We have

$$\begin{aligned}P\Big(\boldsymbol{x}^{(j)},\boldsymbol{m}^{(j)}\,\big|\,\boldsymbol{\theta}\Big) =& P\Big(\boldsymbol{y}^{(j)},\boldsymbol{x}^{(j)},\boldsymbol{m}^{(j)},m^{(j)}\big|\boldsymbol{\theta}\Big)\\ =& P\Big(\boldsymbol{x}^{(j)}\,\big|\boldsymbol{y}^{(j)},\boldsymbol{m}^{(j)},m^{(j)},\boldsymbol{\theta}\Big)P\Big(\boldsymbol{y}^{(j)},\boldsymbol{m}^{(j)},m^{(j)}\big|\boldsymbol{\theta}\Big).\end{aligned} \tag{88}$$

Because $\boldsymbol{y}^{(j)}$ fully determines $\boldsymbol{x}^{(j)}$, we have

$$P\Big(\boldsymbol{x}^{(j)}\,\big|\boldsymbol{y}^{(j)},\boldsymbol{m}^{(j)},m^{(j)},\boldsymbol{\theta}\Big) = P\Big(\boldsymbol{x}^{(j)}\,\big|\boldsymbol{y}^{(j)},\boldsymbol{\theta}\Big). \tag{89}$$

Furthermore,

$$P\Big(\boldsymbol{y}^{(j)},\boldsymbol{m}^{(j)},m^{(j)}\big|\boldsymbol{\theta}\Big) = P\Big(\boldsymbol{y}^{(j)},\boldsymbol{m}^{(j)}\,\big|m^{(j)},\boldsymbol{\theta}\Big)P\Big(m^{(j)}\big|\boldsymbol{\theta}\Big). \tag{90}$$

Now since $\boldsymbol{y}^{(j)} := \operatorname{sign}\left(\boldsymbol{m}^{(j)}\right)$ we have

$$P\left(\boldsymbol{y}^{(j)},\boldsymbol{m}^{(j)}\,\middle|\,m^{(j)},\boldsymbol{\theta}\right) = P\left(\boldsymbol{m}^{(j)}\,\middle|\,m^{(j)},\boldsymbol{\theta}\right). \tag{91}$$

Thus, we can rewrite (88) as

$$P\left(\boldsymbol{m}^{(j)},\boldsymbol{x}^{(j)}\,\middle|\,\boldsymbol{\theta}\right) = P\left(\boldsymbol{x}^{(j)}\,\middle|\,\boldsymbol{y}^{(j)},\boldsymbol{\theta}\right)P\left(\boldsymbol{m}^{(j)}\,\middle|\,m^{(j)},\boldsymbol{\theta}\right)P\left(m^{(j)}\middle|\boldsymbol{\theta}\right). \tag{92}$$

The first term on the right-hand side is the probability of observed data $\boldsymbol{x}^{(j)}$ given that the true absence/presence of lineages are $\boldsymbol{y}^{(j)}$ (6). The second term is the probability that $m_k^{(j)}$ of the infecting lineages within the infection is the lineage A_k given that the MOI is $m^{(j)}$. This is a multinomial distribution and is given by

$$P\left(\boldsymbol{m}^{(j)}\,\middle|\,m^{(j)},\boldsymbol{\theta}\right) = \binom{m^{(j)}}{\boldsymbol{m}^{(j)}}p_1^{m_1^{(j)}}\,\dots\,p_n^{m_n^{(j)}},$$

(cf. [4]). The third term is the probability of MOI m which is a conditional Poisson distribution and is specified in (1). Therefore, (92) becomes

$$P\left(\boldsymbol{x}^{(j)},\boldsymbol{y}^{(j)},\boldsymbol{m}^{(j)}\,\middle|\,\boldsymbol{\theta}\right) = \frac{1}{e^{\lambda}-1}\frac{\lambda^{m^{(j)}}}{m^{(j)}!}\binom{m^{(j)}}{\boldsymbol{m}^{(j)}}\prod_{k=1}^{n}\left(p_k^{m_k^{(j)}}(1-\varepsilon)^{x_k^{(j)}}\varepsilon^{y_k^{(j)}-x_k^{(j)}}\right). \tag{93}$$

Thus, rewriting (87) by using (93) gives

$$\begin{aligned}
Q_j\left(\boldsymbol{\theta}\,|\,\boldsymbol{\theta}^{(t)}\right) =&\,\mathbb{E}_{\boldsymbol{m}^{(j)}\,|\,\boldsymbol{x}^{(j)},\boldsymbol{\theta}^{(t)}}\Bigg(-\log\left(e^{\lambda}-1\right)+m^{(j)}\log(\lambda)-\sum_{k=1}^{n}\log\left(m_k^{(j)}!\right)\\
&+\sum_{k=1}^{n}m_k^{(j)}\log(p_k)+\log(1-\varepsilon)\sum_{k=1}^{n}x_k^{(j)}+\log(\varepsilon)\sum_{k=1}^{n}\left(y_k^{(j)}-x_k^{(j)}\right)\Bigg)\\
=&C_j^{(t)}-\log\left(e^{\lambda}-1\right)+\log(\lambda)\,\mathbb{E}_{\boldsymbol{m}^{(j)}\,|\,\boldsymbol{x}^{(j)},\boldsymbol{\theta}^{(t)}}\left(m^{(j)}\right) \qquad (94a)\\
&+\sum_{k=1}^{n}\log(p_k)\,\mathbb{E}_{\boldsymbol{m}^{(j)}\,|\,\boldsymbol{x}^{(j)},\boldsymbol{\theta}^{(t)}}\left(m_k^{(j)}\right)\\
&+\log(1-\varepsilon)\sum_{k=1}^{n}x_k+\log(\varepsilon)\,\mathbb{E}_{\boldsymbol{m}^{(j)}\,|\,\boldsymbol{x}^{(j)},\boldsymbol{\theta}^{(t)}}\left(\sum_{k=1}^{n}\left(y_k^{(j)}-x_k^{(j)}\right)\right),
\end{aligned}$$

where

$$C_j^{(t)} = -\,\mathbb{E}_{\boldsymbol{m}^{(j)}\,|\,\boldsymbol{x}^{(j)},\boldsymbol{\theta}^{(t)}}\left(\sum_{k=1}^{n}\log\left(m_k^{(j)}!\right)\right), \tag{94b}$$

is independent of $\boldsymbol{\theta}$.

To simplify $Q_j(\boldsymbol{\theta}\,|\,\boldsymbol{\theta}^{(t)})$ the conditional expectation needs to be calculated explicitly. For the following calculations, we drop the super scripts j and consider $\boldsymbol{x}$ to be an arbitrary non-empty record in $\mathcal{X}$. Following the definition of conditional expectation, we derive

$$\mathbb{E}_{\boldsymbol{m}\,|\,\boldsymbol{x},\boldsymbol{\theta}^{(t)}}\left(m_k\right) = \sum_{\boldsymbol{m}}m_kP\left(\boldsymbol{m}\,|\,\boldsymbol{x},\boldsymbol{\theta}^{(t)}\right) = \frac{1}{P\left(\boldsymbol{x}\,|\,\boldsymbol{\theta}^{(t)}\right)}\sum_{\boldsymbol{m}}m_kP\left(\boldsymbol{m},\boldsymbol{x}\,|\,\boldsymbol{\theta}^{(t)}\right), \tag{95a}$$

$$\mathbb{E}_{\boldsymbol{m}\,|\,\boldsymbol{x},\boldsymbol{\theta}^{(t)}}\left(m\right) = \sum_{k=1}^{n}\mathbb{E}_{\boldsymbol{m}\,|\,\boldsymbol{x},\boldsymbol{\theta}^{(t)}}\left(m_k\right) \tag{95b}$$

and

$$\mathbb{E}_{\boldsymbol{m}\,|\,\boldsymbol{x},\boldsymbol{\theta}^{(t)}}\Big(\sum_{k=1}^{n}(y_k-x_k)\Big)=\sum_{\boldsymbol{m}}\Big(\sum_{k=1}^{n}(y_k-x_k)\Big)P\big(\boldsymbol{m}\,|\,\boldsymbol{x},\boldsymbol{\theta}^{(t)}\big)$$
$$=\frac{1}{P\big(\boldsymbol{x}\,|\,\boldsymbol{\theta}^{(t)}\big)}\sum_{\boldsymbol{m}}\Big(\sum_{k=1}^{n}(y_k-x_k)\Big)P\big(\boldsymbol{m},\boldsymbol{x}\,|\,\boldsymbol{\theta}^{(t)}\big). \tag{95c}$$

By rearranging (6) we obtain

$$P\big(\boldsymbol{x}\,|\,\boldsymbol{\theta}^{(t)}\big)=\frac{\big(1-\varepsilon^{(t)}\big)^{|\boldsymbol{x}|}}{e^{\lambda^{(t)}}-1}\prod_{k=1}^{n}\Big(e^{\lambda^{(t)}p_k^{(t)}}-1\Big)^{x_k}\Big(\varepsilon^{(t)}(e^{\lambda^{(t)}p_k^{(t)}}-1)+1\Big)^{1-x_k}. \tag{96}$$

Let

$$S_1:=\sum_{\boldsymbol{m}}mP\big(\boldsymbol{m},\boldsymbol{x}\,|\,\boldsymbol{\theta}^{(t)}\big)$$
$$=\sum_{\boldsymbol{x}\le\boldsymbol{y}}\sum_{m=|\boldsymbol{y}|}^{\infty}m\,\kappa_m^{(t)}\sum_{\substack{\boldsymbol{m}:\\ \operatorname{sign}\boldsymbol{m}=\boldsymbol{y}\\ |\boldsymbol{m}|=m}}\binom{m}{\boldsymbol{m}}\boldsymbol{p}^{(t)\boldsymbol{m}}\prod_{k=1}^{n}\big(1-\varepsilon^{(t)}\big)^{x_k}\varepsilon^{(t)(y_k-x_k)}, \tag{97a}$$
$$S_2:=\sum_{\boldsymbol{m}}m_kP\big(\boldsymbol{m},\boldsymbol{x}\,|\,\boldsymbol{\theta}^{(t)}\big)$$
$$=\sum_{\boldsymbol{x}\le\boldsymbol{y}}\sum_{m=|\boldsymbol{y}|}^{\infty}\kappa_m^{(t)}\sum_{\substack{\boldsymbol{m}:\\ \operatorname{sign}\boldsymbol{m}=\boldsymbol{y}\\ |\boldsymbol{m}|=m}}m_k\binom{m}{\boldsymbol{m}}\boldsymbol{p}^{(t)\boldsymbol{m}}\prod_{k=1}^{n}\big(1-\varepsilon^{(t)}\big)^{x_k}\varepsilon^{(t)(y_k-x_k)}, \tag{97b}$$

and

$$S_3:=\sum_{\boldsymbol{m}}\Big(\sum_{k=1}^{n}(y_k-x_k)\Big)P\big(\boldsymbol{m},\boldsymbol{x}\,|\,\boldsymbol{\theta}^{(t)}\big)$$
$$=\sum_{\boldsymbol{x}\le\boldsymbol{y}}\sum_{k=1}^{n}(y_k-x_k)\sum_{m=|\boldsymbol{y}|}^{\infty}\kappa_m^{(t)}\sum_{\substack{\boldsymbol{m}:\\ \operatorname{sign}\boldsymbol{m}=\boldsymbol{y}\\ |\boldsymbol{m}|=m}}\binom{m}{\boldsymbol{m}}\boldsymbol{p}^{(t)\boldsymbol{m}}\prod_{k=1}^{n}\big(1-\varepsilon^{(t)}\big)^{x_k}\varepsilon^{(t)(y_k-x_k)}. \tag{97c}$$

We can rewrite S_1 as

$$S_1=\frac{\big(1-\varepsilon^{(t)}\big)^{|\boldsymbol{x}|}}{e^{\lambda^{(t)}}-1}\sum_{\boldsymbol{x}\le\boldsymbol{y}}\varepsilon^{(t)|\boldsymbol{y}|-|\boldsymbol{x}|}\lambda^{(t)}\frac{\partial}{\partial\lambda^{(t)}}\Bigg(\sum_{m=|\boldsymbol{y}|}^{\infty}\frac{\lambda^{(t)m}}{m!}\sum_{\substack{\boldsymbol{m}:\\ \operatorname{sign}\boldsymbol{m}=\boldsymbol{y}\\ |\boldsymbol{m}|=m}}\binom{m}{\boldsymbol{m}}\boldsymbol{p}^{(t)\boldsymbol{m}}\Bigg)$$
$$=\frac{\big(1-\varepsilon^{(t)}\big)^{|\boldsymbol{x}|}}{e^{\lambda^{(t)}}-1}\sum_{\boldsymbol{x}\le\boldsymbol{y}}\varepsilon^{(t)|\boldsymbol{y}|-|\boldsymbol{x}|}\lambda^{(t)}\frac{\partial}{\partial\lambda^{(t)}}\Bigg(\sum_{m=|\boldsymbol{y}|}^{\infty}\sum_{\substack{\boldsymbol{m}:\\ \operatorname{sign}\boldsymbol{m}=\boldsymbol{y}\\ |\boldsymbol{m}|=m}}\prod_{k=1}^{n}\frac{(\lambda^{(t)}p_k^{(t)})^{m_k}}{m_k!}\Bigg)$$
$$=\frac{\big(1-\varepsilon^{(t)}\big)^{|\boldsymbol{x}|}}{e^{\lambda^{(t)}}-1}\sum_{\boldsymbol{x}\le\boldsymbol{y}}\varepsilon^{(t)|\boldsymbol{y}|-|\boldsymbol{x}|}\lambda^{(t)}\frac{\partial}{\partial\lambda^{(t)}}\Bigg(\prod_{\substack{k=1\\ y_k=1}}^{n}\sum_{i=1}^{\infty}\frac{(\lambda^{(t)}p_k^{(t)})^{i}}{i!}\Bigg)$$
$$=\frac{\big(1-\varepsilon^{(t)}\big)^{|\boldsymbol{x}|}}{e^{\lambda^{(t)}}-1}\sum_{\boldsymbol{x}\le\boldsymbol{y}}\varepsilon^{(t)|\boldsymbol{y}|-|\boldsymbol{x}|}\lambda^{(t)}\frac{\partial}{\partial\lambda^{(t)}}\Bigg(\prod_{k=1}^{n}\big(e^{\lambda^{(t)}p_k^{(t)}}-1\big)^{y_k}\Bigg)$$
$$=\frac{\big(1-\varepsilon^{(t)}\big)^{|\boldsymbol{x}|}}{e^{\lambda^{(t)}}-1}\lambda^{(t)}\sum_{\boldsymbol{x}\le\boldsymbol{y}}\varepsilon^{(t)|\boldsymbol{y}|-|\boldsymbol{x}|}\Bigg(\sum_{k=1}^{n}\frac{y_kp_k^{(t)}}{1-e^{-\lambda^{(t)}p_k^{(t)}}}\Bigg)\prod_{k=1}^{n}\big(e^{\lambda^{(t)}p_k^{(t)}}-1\big)^{y_k}.$$

As above (2), let $\mathcal{A}(\boldsymbol{x}) = \{k \,|\, x_k = 0\}$. We can rewrite S_1 as

$$S_1 = \frac{\lambda^{(t)}\big(1-\varepsilon^{(t)}\big)^{|\boldsymbol{x}|}}{e^{\lambda^{(t)}}-1}\prod_{k=1}^{n}\Big(e^{\lambda^{(t)}p_k^{(t)}}-1\Big)^{x_k} S_1^*,$$

where

$$S_1^* = \left(\sum_{\mathcal{F}\subseteq\mathcal{A}(\boldsymbol{x})}\left(\sum_{k=1}^{n}\frac{x_k p_k^{(t)}}{1-e^{-\lambda^{(t)}p_k^{(t)}}} + \sum_{h\in\mathcal{F}}\frac{p_h^{(t)}}{1-e^{-\lambda^{(t)}p_h^{(t)}}}\right)\prod_{h\in\mathcal{F}}\Big(\varepsilon^{(t)}(e^{\lambda^{(t)}p_h^{(t)}}-1)\Big)\right).$$

Separating the other sum and using the identity

$$\sum_{\mathcal{F}\subseteq\mathcal{A}(\boldsymbol{x})}\prod_{h\in\mathcal{F}} a_h = \prod_{h\in\mathcal{A}(\boldsymbol{x})}(a_h+1) \tag{98}$$

for $a_h = \varepsilon^{(t)}(e^{\lambda^{(t)}p_h^{(t)}}-1)$ gives

$$\begin{aligned}
S_1^* =& \Bigg(\left(\sum_{k=1}^{n}\frac{x_k p_k^{(t)}}{1-e^{-\lambda^{(t)}p_k^{(t)}}}\right)\prod_{k=1}^{n}\Big(\varepsilon^{(t)}(e^{\lambda^{(t)}p_k^{(t)}}-1)+1\Big)^{1-x_k} \\
&+ \sum_{\mathcal{F}\subseteq\mathcal{A}(\boldsymbol{x})}\left(\sum_{h\in\mathcal{F}}\frac{p_h^{(t)}}{1-e^{-\lambda^{(t)}p_h^{(t)}}}\right)\prod_{h\in\mathcal{F}}\Big(\varepsilon^{(t)}(e^{\lambda^{(t)}p_h^{(t)}}-1)\Big)\Bigg) \\
=& \Bigg(\left(\sum_{k=1}^{n}\frac{x_k p_k^{(t)}}{1-e^{-\lambda^{(t)}p_k^{(t)}}}\right)\prod_{k=1}^{n}\Big(\varepsilon^{(t)}(e^{\lambda^{(t)}p_k^{(t)}}-1)+1\Big)^{1-x_k} \\
&+ \frac{\partial}{\partial\lambda^{(t)}}\left(\sum_{\mathcal{F}\subseteq\mathcal{A}(\boldsymbol{x})}\prod_{h\in\mathcal{F}}\Big(\varepsilon^{(t)}(e^{\lambda^{(t)}p_h^{(t)}}-1)\Big)\right)\Bigg).
\end{aligned}$$

Using the identity (98) in the last term and taking the derivative yields

$$\begin{aligned}
S_1^* =& \Bigg(\left(\sum_{k=1}^{n}\frac{x_k p_k^{(t)}}{1-e^{-\lambda^{(t)}p_k^{(t)}}}\right)\prod_{k=1}^{n}\Big(\varepsilon^{(t)}(e^{\lambda^{(t)}p_k^{(t)}}-1)+1\Big)^{1-x_k} \\
&+ \left(\sum_{k=1}^{n}\frac{(1-x_k)\varepsilon^{(t)}p_k^{(t)}e^{\lambda^{(t)}p_k^{(t)}}}{\varepsilon^{(t)}(e^{\lambda^{(t)}p_k^{(t)}}-1)+1}\right)\prod_{k=1}^{n}\Big(\varepsilon^{(t)}(e^{\lambda^{(t)}p_k^{(t)}}-1)+1\Big)^{1-x_k}\Bigg) \\
=& \prod_{k=1}^{n}\Big(\varepsilon^{(t)}(e^{\lambda^{(t)}p_k^{(t)}}-1)+1\Big)^{1-x_k}\left(\sum_{k=1}^{n}\frac{x_k p_k^{(t)}}{1-e^{-\lambda^{(t)}p_k^{(t)}}} + \frac{(1-x_k)\varepsilon^{(t)}p_k^{(t)}e^{\lambda^{(t)}p_k^{(t)}}}{\varepsilon^{(t)}(e^{\lambda^{(t)}p_k^{(t)}}-1)+1}\right).
\end{aligned}$$

Therefore, S_1 becomes

$$\begin{aligned}
S_1 =& \frac{\lambda^{(t)}\big(1-\varepsilon^{(t)}\big)^{|\boldsymbol{x}|}}{e^{\lambda^{(t)}}-1}\prod_{k=1}^{n}\Big(e^{\lambda^{(t)}p_k^{(t)}}-1\Big)^{x_k}\Big(\varepsilon^{(t)}(e^{\lambda^{(t)}p_k^{(t)}}-1)+1\Big)^{1-x_k} \\
&\left(\sum_{k=1}^{n}\frac{x_k p_k^{(t)}}{1-e^{-\lambda^{(t)}p_k^{(t)}}} + \frac{(1-x_k)\varepsilon^{(t)}p_k^{(t)}e^{\lambda^{(t)}p_k^{(t)}}}{\varepsilon^{(t)}(e^{\lambda^{(t)}p_k^{(t)}}-1)+1}\right).
\end{aligned}$$

Similar calculations give

$$
\begin{aligned}
S_2 &= \sum_{\boldsymbol{x}\leq\boldsymbol{y}} \sum_{m=|\boldsymbol{y}|}^{\infty} \kappa_m^{(t)} \sum_{\substack{\boldsymbol{m}:\\ \operatorname{sign}\boldsymbol{m}=\boldsymbol{y}\\ |\boldsymbol{m}|=m}} m_k \binom{m}{\boldsymbol{m}} \boldsymbol{p}^{(t)\boldsymbol{m}} \prod_{k=1}^{n} \left(1-\varepsilon^{(t)}\right)^{x_k} \varepsilon^{(t)(y_k-x_k)} \\
&= \frac{\left(1-\varepsilon^{(t)}\right)^{|\boldsymbol{x}|}}{e^{\lambda^{(t)}}-1} p_k^{(t)} \frac{\partial}{\partial p_k^{(t)}} \left(\sum_{\boldsymbol{x}\leq\boldsymbol{y}} \varepsilon^{(t)|\boldsymbol{y}|-|\boldsymbol{x}|} \sum_{m=|\boldsymbol{y}|}^{\infty} \frac{\lambda^{(t)m}}{m!} \sum_{\substack{\boldsymbol{m}:\\ \operatorname{sign}\boldsymbol{m}=\boldsymbol{y}\\ |\boldsymbol{m}|=m}} \binom{m}{\boldsymbol{m}} \boldsymbol{p}^{(t)\boldsymbol{m}} \right) \\
&= \frac{\left(1-\varepsilon^{(t)}\right)^{|\boldsymbol{x}|}}{e^{\lambda^{(t)}}-1} p_k^{(t)} \frac{\partial}{\partial p_k^{(t)}} \left(\prod_{k=1}^{n} \left(e^{\lambda^{(t)}p_k^{(t)}}-1\right)^{x_k} \left(\varepsilon^{(t)}(e^{\lambda^{(t)}p_k^{(t)}}-1)+1\right)^{1-x_k} \right) \\
&= \frac{p_k^{(t)}\left(1-\varepsilon^{(t)}\right)^{|\boldsymbol{x}|}}{e^{\lambda^{(t)}}-1} \prod_{k=1}^{n} \left(e^{\lambda^{(t)}p_k^{(t)}}-1\right)^{x_k} \left(\varepsilon^{(t)}(e^{\lambda^{(t)}p_k^{(t)}}-1)+1\right)^{1-x_k} \\
&\qquad \left(x_k \frac{\lambda^{(t)}}{1-e^{-\lambda^{(t)}p_k^{(t)}}} + (1-x_k) \frac{\lambda^{(t)}\varepsilon^{(t)} e^{\lambda^{(t)}p_k^{(t)}}}{\varepsilon^{(t)}(e^{\lambda^{(t)}p_k^{(t)}}-1)+1} \right).
\end{aligned}
$$

Finally, similar calculations yield

$$
\begin{aligned}
S_3 &= \sum_{\boldsymbol{x}\leq\boldsymbol{y}} \sum_{k=1}^{n} (y_k-x_k) \sum_{m=|\boldsymbol{y}|}^{\infty} \kappa_m^{(t)} \sum_{\substack{\boldsymbol{m}:\\ \operatorname{sign}\boldsymbol{m}=\boldsymbol{y}\\ |\boldsymbol{m}|=m}} \binom{m}{\boldsymbol{m}} \boldsymbol{p}^{(t)\boldsymbol{m}} \prod_{k=1}^{n} \left(1-\varepsilon^{(t)}\right)^{x_k} \varepsilon^{(t)(y_k-x_k)} \\
&= \frac{\left(1-\varepsilon^{(t)}\right)^{|\boldsymbol{x}|}}{e^{\lambda^{(t)}}-1} \sum_{\boldsymbol{x}\leq\boldsymbol{y}} (|\boldsymbol{y}|-|\boldsymbol{x}|) \varepsilon^{(t)|\boldsymbol{y}|-|\boldsymbol{x}|} \sum_{m=|\boldsymbol{y}|}^{\infty} \frac{\lambda^{(t)m}}{m!} \sum_{\substack{\boldsymbol{m}:\\ \operatorname{sign}\boldsymbol{m}=\boldsymbol{y}\\ |\boldsymbol{m}|=m}} \binom{m}{\boldsymbol{m}} \boldsymbol{p}^{(t)\boldsymbol{m}},
\end{aligned}
$$

where

$$
\sum_{m=|\boldsymbol{y}|}^{\infty} \frac{\lambda^{(t)m}}{m!} \sum_{\substack{\boldsymbol{m}:\\ \operatorname{sign}\boldsymbol{m}=\boldsymbol{y}\\ |\boldsymbol{m}|=m}} \binom{m}{\boldsymbol{m}} \boldsymbol{p}^{(t)\boldsymbol{m}} = \prod_{k=1}^{n} \left(e^{\lambda^{(t)}p_k^{(t)}}-1\right)^{y_k},
$$

(for a proof of the latter see [4]). Hence,

$$
\begin{aligned}
S_3 &= \frac{\left(1-\varepsilon^{(t)}\right)^{|\boldsymbol{x}|}}{e^{\lambda^{(t)}}-1} \sum_{\boldsymbol{x}\leq\boldsymbol{y}} (|\boldsymbol{y}|-|\boldsymbol{x}|) \varepsilon^{(t)|\boldsymbol{y}|-|\boldsymbol{x}|} \prod_{k=1}^{n} (e^{\lambda^{(t)}p_k^{(t)}}-1)^{y_k} \\
&= \frac{\left(1-\varepsilon^{(t)}\right)^{|\boldsymbol{x}|}}{e^{\lambda^{(t)}}-1} \prod_{k=1}^{n} \left(e^{\lambda^{(t)}p_k^{(t)}}-1\right)^{x_k} \sum_{\mathcal{F}\subseteq\mathcal{A}(\boldsymbol{x})} |\mathcal{F}| \varepsilon^{(t)|\mathcal{F}|} \prod_{h\in\mathcal{F}} (e^{\lambda^{(t)}p_h^{(t)}}-1) \\
&= \frac{\varepsilon^{(t)}\left(1-\varepsilon^{(t)}\right)^{|\boldsymbol{x}|}}{e^{\lambda^{(t)}}-1} \prod_{k=1}^{n} \left(e^{\lambda^{(t)}p_k^{(t)}}-1\right)^{x_k} \frac{\partial}{\partial \varepsilon^{(t)}} \left(\sum_{\mathcal{F}\subseteq\mathcal{A}(\boldsymbol{x})} \prod_{h\in\mathcal{F}} \left(\varepsilon^{(t)}(e^{\lambda^{(t)}p_h^{(t)}}-1)\right) \right) \\
&= \frac{\varepsilon^{(t)}\left(1-\varepsilon^{(t)}\right)^{|\boldsymbol{x}|}}{e^{\lambda^{(t)}}-1} \prod_{k=1}^{n} \left(e^{\lambda^{(t)}p_k^{(t)}}-1\right)^{x_k} \frac{\partial}{\partial \varepsilon^{(t)}} \left(\prod_{k=1}^{n} \left(\varepsilon^{(t)}(e^{\lambda^{(t)}p_k^{(t)}}-1)+1\right)^{1-x_k} \right) \\
&= \frac{\varepsilon^{(t)}\left(1-\varepsilon^{(t)}\right)^{|\boldsymbol{x}|}}{e^{\lambda^{(t)}}-1} \\
&\quad \times \prod_{k=1}^{n} \left(e^{\lambda^{(t)}p_k^{(t)}}-1\right)^{x_k} \left(\varepsilon^{(t)}(e^{\lambda^{(t)}p_k^{(t)}}-1)+1\right)^{1-x_k} \left(\sum_{k=1}^{n} \frac{(1-x_k)(e^{\lambda^{(t)}p_k^{(t)}}-1)}{\varepsilon^{(t)}(e^{\lambda^{(t)}p_h^{(t)}}-1)+1} \right).
\end{aligned}
$$

Therefore, (95) becomes

$$\mathbb{E}_{\boldsymbol{m}\,|\,\boldsymbol{x},\boldsymbol{\theta}^{(t)}}\left(m_k\right) = p_k^{(t)}\left(x_k \frac{\lambda^{(t)}}{1-e^{-\lambda^{(t)}p_k^{(t)}}} + (1-x_k)\frac{\lambda^{(t)}\varepsilon^{(t)}e^{\lambda^{(t)}p_k^{(t)}}}{\varepsilon^{(t)}(e^{\lambda^{(t)}p_k^{(t)}}-1)+1}\right), \tag{100a}$$

$$\mathbb{E}_{\boldsymbol{m}\,|\,\boldsymbol{x},\boldsymbol{\theta}^{(t)}}\left(m\right) = \lambda^{(t)}\left(\sum_{k=1}^{n} x_k \frac{p_k^{(t)}}{1-e^{-\lambda^{(t)}p_k^{(t)}}} + (1-x_k)\frac{\varepsilon^{(t)}p_k^{(t)}e^{\lambda^{(t)}p_k^{(t)}}}{\varepsilon^{(t)}(e^{\lambda^{(t)}p_k^{(t)}}-1)+1}\right), \tag{100b}$$

and

$$\mathbb{E}_{\boldsymbol{m}\,|\,\boldsymbol{x},\boldsymbol{\theta}^{(t)}}\left(\sum_{k=1}^{n}(y_k-x_k)\right) = \varepsilon^{(t)}\left(\sum_{k=1}^{n}\frac{(1-x_k)(e^{\lambda^{(t)}p_k^{(t)}}-1)}{\varepsilon^{(t)}(e^{\lambda^{(t)}p_h^{(t)}}-1)+1}\right). \tag{100c}$$

So far we derived the required terms conditioned on a non-empty record $\boldsymbol{x}$. It is not difficult to see that for an empty record $\boldsymbol{x}=\mathbf{0}$ we have

$$\mathbb{E}_{\boldsymbol{m}\,|\,\mathbf{0},\boldsymbol{\theta}^{(t)}}\left(m_k\right) = \frac{\prod_{k=1}^{n}\left(\varepsilon^{(t)}(e^{\lambda^{(t)}p_k^{(t)}}-1)+1\right)}{-1+\prod_{k=1}^{n}\left(\varepsilon^{(t)}(e^{\lambda^{(t)}p_k^{(t)}}-1)+1\right)} p_k^{(t)}\frac{\lambda^{(t)}\varepsilon^{(t)}e^{\lambda^{(t)}p_k^{(t)}}}{\varepsilon^{(t)}(e^{\lambda^{(t)}p_k^{(t)}}-1)+1}, \tag{101a}$$

$$\mathbb{E}_{\boldsymbol{m}\,|\,\mathbf{0},\boldsymbol{\theta}^{(t)}}\left(m\right) = \frac{\prod_{k=1}^{n}\left(\varepsilon^{(t)}(e^{\lambda^{(t)}p_k^{(t)}}-1)+1\right)}{-1+\prod_{k=1}^{n}\left(\varepsilon^{(t)}(e^{\lambda^{(t)}p_k^{(t)}}-1)+1\right)}\lambda^{(t)}\sum_{k=1}^{n}\frac{\varepsilon^{(t)}p_k^{(t)}e^{\lambda^{(t)}p_k^{(t)}}}{\varepsilon^{(t)}(e^{\lambda^{(t)}p_k^{(t)}}-1)+1}, \tag{101b}$$

and

$$\mathbb{E}_{\boldsymbol{m}\,|\,\mathbf{0},\boldsymbol{\theta}^{(t)}}\left(\sum_{k=1}^{n}y_k\right) = \frac{\prod_{k=1}^{n}\left(\varepsilon^{(t)}(e^{\lambda^{(t)}p_k^{(t)}}-1)+1\right)}{-1+\prod_{k=1}^{n}\left(\varepsilon^{(t)}(e^{\lambda^{(t)}p_k^{(t)}}-1)+1\right)}\varepsilon^{(t)}\sum_{k=1}^{n}\frac{e^{\lambda^{(t)}p_k^{(t)}}-1}{\varepsilon^{(t)}(e^{\lambda^{(t)}p_h^{(t)}}-1)+1}. \tag{101c}$$

Finally, we can rewrite $Q_j(\boldsymbol{\theta}\,|\,\boldsymbol{\theta}^{(t)})$ as

$$\begin{aligned}
Q_j\left(\boldsymbol{\theta}\,|\,\boldsymbol{\theta}^{(t)}\right) =& \lambda^{(t)}\left(\sum_{k=1}^{n} x_k^{(j)}\frac{p_k^{(t)}}{1-e^{-\lambda^{(t)}p_k^{(t)}}} + (1-x_k^{(j)})\frac{\varepsilon^{(t)}p_k^{(t)}e^{\lambda^{(t)}p_k^{(t)}}}{\varepsilon^{(t)}(e^{\lambda^{(t)}p_k^{(t)}}-1)+1}\right)\log(\lambda)\\
&+\sum_{k=1}^{n}p_k^{(t)}\left(x_k^{(j)}\frac{\lambda^{(t)}}{1-e^{-\lambda^{(t)}p_k^{(t)}}} + (1-x_k^{(j)})\frac{\lambda^{(t)}\varepsilon^{(t)}e^{\lambda^{(t)}p_k^{(t)}}}{\varepsilon^{(t)}(e^{\lambda^{(t)}p_k^{(t)}}-1)+1}\right)\log(p_k)\\
&+\varepsilon^{(t)}\left(\sum_{k=1}^{n}\frac{(1-x_k^{(j)})(e^{\lambda^{(t)}p_k^{(t)}}-1)}{\varepsilon^{(t)}(e^{\lambda^{(t)}p_h^{(t)}}-1)+1}\right)\log(\varepsilon) + (1-\varepsilon)\sum_{k=1}^{n}x_k^{(j)}\\
&-\log\left(e^{\lambda}-1\right) + C_j^{(t)},
\end{aligned}$$

for $\boldsymbol{x}^{(j)} \neq \mathbf{0}$, and

$$Q_j\big(\boldsymbol{\theta}|\boldsymbol{\theta}^{(t)}\big) = \frac{\prod_{k=1}^{n}\Big(\varepsilon^{(t)}(e^{\lambda^{(t)}p_k^{(t)}}-1)+1\Big)}{-1+\prod_{k=1}^{n}\Big(\varepsilon^{(t)}(e^{\lambda^{(t)}p_k^{(t)}}-1)+1\Big)}\Bigg(\lambda^{(t)}\Bigg(\sum_{k=1}^{n}\frac{\varepsilon^{(t)}p_k^{(t)}e^{\lambda^{(t)}p_k^{(t)}}}{\varepsilon^{(t)}(e^{\lambda^{(t)}p_k^{(t)}}-1)+1}\Bigg)\log(\lambda)$$
$$+\sum_{k=1}^{n}\Bigg(p_k^{(t)}\frac{\lambda^{(t)}\varepsilon^{(t)}e^{\lambda^{(t)}p_k^{(t)}}}{\varepsilon^{(t)}(e^{\lambda^{(t)}p_k^{(t)}}-1)+1}\log(p_k)\Bigg)$$
$$+\varepsilon^{(t)}\Bigg(\sum_{k=1}^{n}\frac{e^{\lambda^{(t)}p_k^{(t)}}-1}{\varepsilon^{(t)}(e^{\lambda^{(t)}p_h^{(t)}}-1)+1}\Bigg)\log(\varepsilon)\Bigg)$$
$$-\log\big(e^{\lambda}-1\big)+C_j^{(t)},$$

if $\boldsymbol{x}^{(j)} = \mathbf{0}$. Note that equation (86) is derived as

$$Q\big(\boldsymbol{\theta}|\boldsymbol{\theta}^{(t)}\big) = \sum_{j=1}^{N}Q_j\big(\boldsymbol{\theta}|\boldsymbol{\theta}^{(t)}\big) = \sum_{\boldsymbol{x}^{(j)}\neq\mathbf{0}}Q_j\big(\boldsymbol{\theta}|\boldsymbol{\theta}^{(t)}\big)+\sum_{\boldsymbol{x}^{(j)}=\mathbf{0}}Q_j\big(\boldsymbol{\theta}|\boldsymbol{\theta}^{(t)}\big), \tag{102}$$

where the first sum on the right-hand side runs over all non-empty records and the second sum runs over all empty records in $\mathcal{X}$. By using $N_k = \sum_{j=1}^{n}x_k^{(j)}$, and that N_+ and $n_{\mathbf{0}}$ are respectively the number of non-empty and empty records, we derive

$$\sum_{\boldsymbol{x}^{(j)}\neq\mathbf{0}}Q_j\big(\boldsymbol{\theta}|\boldsymbol{\theta}^{(t)}\big) = \lambda^{(t)}\Bigg(\sum_{k=1}^{n}N_k\frac{p_k^{(t)}}{1-e^{-\lambda^{(t)}p_k^{(t)}}}+(N_+-N_k)\frac{\varepsilon^{(t)}p_k^{(t)}e^{\lambda^{(t)}p_k^{(t)}}}{\varepsilon^{(t)}\big(e^{\lambda^{(t)}p_k^{(t)}}-1\big)+1}\Bigg)\log(\lambda)$$
$$+\sum_{k=1}^{n}p_k^{(t)}\Bigg(N_k\frac{\lambda^{(t)}}{1-e^{-\lambda^{(t)}p_k^{(t)}}}+(N_+-N_k)\frac{\lambda^{(t)}\varepsilon^{(t)}e^{\lambda^{(t)}p_k^{(t)}}}{\varepsilon^{(t)}\big(e^{\lambda^{(t)}p_k^{(t)}}-1\big)+1}\Bigg)\log(p_k)$$
$$+\log\big(1-\varepsilon\big)\sum_{k=1}^{n}N_k+\varepsilon^{(t)}\Bigg(\sum_{k=1}^{n}(N_+-N_k)\frac{e^{\lambda^{(t)}p_k^{(t)}}-1}{\varepsilon^{(t)}\big(e^{\lambda^{(t)}p_h^{(t)}}-1\big)+1}\Bigg)\log(\varepsilon)$$
$$-N_+\log\big(e^{\lambda}-1\big)+D_1^{(t)},$$

$$\sum_{\boldsymbol{x}^{(j)}=\mathbf{0}}Q_j\big(\boldsymbol{\theta}|\boldsymbol{\theta}^{(t)}\big) = n_{\mathbf{0}}\frac{\prod_{k=1}^{n}\Big(\varepsilon^{(t)}(e^{\lambda^{(t)}p_k^{(t)}}-1)+1\Big)}{-1+\prod_{k=1}^{n}\Big(\varepsilon^{(t)}(e^{\lambda^{(t)}p_k^{(t)}}-1)+1\Big)}$$
$$\times\Bigg(\lambda^{(t)}\Bigg(\sum_{k=1}^{n}\frac{\varepsilon^{(t)}p_k^{(t)}e^{\lambda^{(t)}p_k^{(t)}}}{\varepsilon^{(t)}(e^{\lambda^{(t)}p_k^{(t)}}-1)+1}\Bigg)\log(\lambda)$$
$$+\sum_{k=1}^{n}\Bigg(p_k^{(t)}\frac{\lambda^{(t)}\varepsilon^{(t)}e^{\lambda^{(t)}p_k^{(t)}}}{\varepsilon^{(t)}(e^{\lambda^{(t)}p_k^{(t)}}-1)+1}\log(p_k)\Bigg)$$
$$+\varepsilon^{(t)}\Bigg(\sum_{k=1}^{n}\frac{e^{\lambda^{(t)}p_k^{(t)}}-1}{\varepsilon^{(t)}(e^{\lambda^{(t)}p_h^{(t)}}-1)+1}\Bigg)\log(\varepsilon)\Bigg)$$
$$-n_{\mathbf{0}}\log\big(e^{\lambda}-1\big)+D_2^{(t)},$$

where $D_1^{(t)} := \sum_{j:\boldsymbol{x}^{(j)}\neq\mathbf{0}}C_j^{(t)}$, $D_2^{(t)} := \sum_{j:\boldsymbol{x}^{(j)}=\mathbf{0}}C_j^{(t)}$ and $D_1^{(t)}+D_2^{(t)} = \sum_{j=1}^{N}C_j^{(t)}$. Further note

$\sum_{j=1}^{N} C_j^{(t)} = D_1^{(t)} + D_2^{(t)}$. Finally, replacing these in (102) gives

$$Q\big(\boldsymbol{\theta}\,|\,\boldsymbol{\theta}^{(t)}\big) = -N\log\big(e^{\lambda}-1\big) + W^{(t)}\log(\lambda) + \sum_{k=1}^{n} U_k^{(t)}\log(p_k) + \log\big(1-\varepsilon\big)\sum_{k=1}^{n} N_k + V^{(t)}\log(\varepsilon) + D^{(t)}, \tag{103}$$

where $D^{(t)} = D_1^{(t)} + D_2^{(t)}$ is a constant and independent of $\boldsymbol{\theta}$, and

$$W^{(t)} = \lambda^{(t)}\Bigg(\Bigg(\sum_{k=1}^{n}\frac{N_k p_k^{(t)}}{1-e^{-\lambda^{(t)}p_k^{(t)}}} + \frac{(N-N_k)\varepsilon^{(t)}p_k^{(t)}e^{\lambda^{(t)}p_k^{(t)}}}{\varepsilon^{(t)}\big(e^{\lambda^{(t)}p_k^{(t)}}-1\big)+1}\Bigg) + T^{(t)}\sum_{k=1}^{n}\frac{\varepsilon^{(t)}p_k^{(t)}e^{\lambda^{(t)}p_k^{(t)}}}{\varepsilon^{(t)}\big(e^{\lambda^{(t)}p_k^{(t)}}-1\big)+1}\Bigg), \tag{104a}$$

$$U_k^{(t)} = \lambda^{(t)}p_k^{(t)}\Bigg(\Bigg(\frac{N_k}{1-e^{-\lambda^{(t)}p_k^{(t)}}} + \frac{(N-N_k)\varepsilon^{(t)}e^{\lambda^{(t)}p_k^{(t)}}}{\varepsilon^{(t)}\big(e^{\lambda^{(t)}p_k^{(t)}}-1\big)+1}\Bigg) + T^{(t)}\frac{\varepsilon^{(t)}e^{\lambda^{(t)}p_k^{(t)}}}{\varepsilon^{(t)}\big(e^{\lambda^{(t)}p_k^{(t)}}-1\big)+1}\Bigg), \tag{104b}$$

$$V^{(t)} = \varepsilon^{(t)}\Bigg(\Bigg(\sum_{k=1}^{n}(N-N_k)\frac{e^{\lambda^{(t)}p_k^{(t)}}-1}{\varepsilon^{(t)}\big(e^{\lambda^{(t)}p_h^{(t)}}-1\big)+1}\Bigg) + T^{(t)}\sum_{k=1}^{n}\frac{e^{\lambda^{(t)}p_k^{(t)}}-1}{\varepsilon^{(t)}\big(e^{\lambda^{(t)}p_h^{(t)}}-1\big)+1}\Bigg), \tag{104c}$$

and

$$T^{(t)} = \frac{n_{\mathbf{0}}}{-1+\prod_{k=1}^{n}\Big(\varepsilon^{(t)}\big(e^{\lambda^{(t)}p_k^{(t)}}-1\big)+1\Big)}. \tag{104d}$$

This concludes the E-step of the algorithm.

Maximization step

The second step of the EM algorithm is carried out by solving

$$\boldsymbol{\theta}^{(t+1)} = \operatorname*{arg\,max}_{\boldsymbol{\theta}\in\Theta} Q\big(\boldsymbol{\theta}\,|\,\boldsymbol{\theta}^{(t)}\big), \tag{105}$$

which is a constrained optimization problem, with the objective function $Q\big(\boldsymbol{\theta}\,|\,\boldsymbol{\theta}^{(t)}\big)$ and the constraint $\sum_{k=1}^{n} p_k = 1$. To find the maximum, introduce a Lagrange multiplier.

Let

$$K\big(\boldsymbol{\theta}\,|\,\boldsymbol{\theta}^{(t)}\big) := Q\big(\boldsymbol{\theta}\,|\,\boldsymbol{\theta}^{(t)}\big) + \gamma\big(1-\sum_{k=1}^{n} p_k\big). \tag{106}$$

Therefore, $\boldsymbol{\theta}^{(t+1)}$ is derived by solving $\nabla K(\boldsymbol{\theta}\,|\,\boldsymbol{\theta}^{(t)}) = 0$, which yields the following system of equations

$$\frac{\partial K}{\partial\lambda}\Big|_{\lambda=\lambda^{(t+1)}} = -\frac{N}{1-e^{-\lambda^{(t+1)}}} + \frac{W^{(t)}}{\lambda^{(t+1)}} = 0, \tag{107a}$$

$$\frac{\partial K}{\partial\varepsilon}\Big|_{\varepsilon=\varepsilon^{(t+1)}} = -\frac{1}{1-\varepsilon^{(t+1)}}\sum_{k=1}^{n} N_k + \frac{V^{(t)}}{\varepsilon^{(t+1)}} = 0, \tag{107b}$$

and

$$\frac{\partial K}{\partial p_k}\Big|_{p_k = p_k^{(t+1)}} = \frac{U_k^{(t)}}{p_k^{(t+1)}} - \gamma = 0. \tag{107c}$$

This system cannot be solved explicitly for all variables. From (107b) and (107c) we have

$$p_k^{(t+1)} = \frac{U_k^{(t)}}{\sum_{k=1}^{n} U_k^{(t)}}, \tag{108a}$$

and

$$\varepsilon^{(t+1)} = \frac{1}{1 + \frac{1}{V^{(t)}} \sum_{k=1}^{n} N_k}, \tag{108b}$$

respectively. We can rewrite (107a) as

$$\lambda^{(t+1)} - \frac{W^{(t)}}{N}\left(1 - e^{-\lambda^{(t+1)}}\right) = 0. \tag{108c}$$

This equation does not permit an explicit solution. However, $\lambda^{(t+1)}$ can easily be derived iteratively by a 1-dimensional Newton method. Let $f(\lambda) = \lambda - \frac{W^{(t)}}{N}\left(1 - e^{-\lambda}\right)$, then the solution to $f(\lambda) = 0$ is found by iterating

$$\lambda_{s+1} = \lambda_s - \frac{\lambda_s - \frac{W^{(t)}}{N}\left(1 - e^{-\lambda_s}\right)}{1 - \frac{W^{(t)}}{N} e^{-\lambda_s}} = \frac{\frac{W^{(t)}}{N}\left(1 - e^{-\lambda_s}(\lambda_s + 1)\right)}{1 - \frac{W^{(t)}}{N} e^{-\lambda_s}}, \tag{108d}$$

which converges from any initial value λ_1 sufficiently larger than $\lambda^{(t+1)}$. The latter follows because $f(0) = 0$ and $f''(\lambda) = \frac{W^{(t)}}{N} e^{-\lambda} > 0$ since $W^{(t)} > 0$ for any t if the data is regular. Therefore, $f(\lambda)$ is strictly convex and finds its minimum at $\log\left(\frac{W^{(t)}}{N}\right)$. The function is monotonically increasing for $\lambda > \log\left(\frac{W^{(t)}}{N}\right)$ and finds its unique solution. Additionally, f''' is continuous, which means the algorithm converges at least at a quadratic rate.

References

1. Schneider KA. Large and finite sample properties of a maximum-likelihood estimator for multiplicity of infection. PLOS ONE. 2018;13(4):1–21. doi:10.1371/journal.pone.0194148.

2. LeVeque WJ. Topics in Number Theory. No. v. 1 in Dover Books on Mathematics. Dover Publications; 2002. Available from: `https://books.google.de/books?id=ocAySqjVLeEC`.

3. Schmidt WM. Diophantine Approximation. Lecture Notes in Mathematics. Springer Berlin Heidelberg; 2009. Available from: `https://books.google.de/books?id=XqZtCQAAQBAJ`.

4. Schneider KA, Escalante AA. A Likelihood Approach to Estimate the Number of Co-Infections. PLoS ONE. 2014;9(7):e97899.
