# Supplementary material for: Estimating multiplicity of infection, allele frequencies, and prevalences accounting for incomplete data
Source: PLoS One. 2024 Mar 21;19(3):e0287161. doi: 10.1371/journal.pone.0287161 (PMC10956774; doi:10.1371/journal.pone.0287161)
Supplement: S2 Data — (PDF) [file pone.0287161.s003.pdf]

Description of R file "MOI-MLE-IDM.R"

Meraj Hashemi, Kristan Schneider

Availability and updates

The R-file "MOI-MLE-IDM.R" is also available via GitHub. Updates of the code and this description will be made available there https://github.com/Maths-against-Malaria/MOI—Incomplete-Data-Model.git. The R-code and description are extensions of those described in Schneider (2018), which is available in an updated version on GitHub https://github.com/Maths-against-Malaria/Maximum-likelihood-estimate-MOI-and-lineage-frequency-distribution.git.

The maximum-likelihood Estimates (MLE)

All functions needed to calculate the MLE of MOI and lineage frequencies from molecular datasets based on the original model (OM) and the incomplete-data model (IDM) are described here.

The first step is to load the R-file "MOI-MLE-IDM.R". The second step is to is to import the data using the function `DatImp`. The third step is to calculate sample size and the prevalence counts for all lineages using the function `Nk`. The final step is to derive the MLE based on the OM or IDM using the function `MLE`.

Loading the R-file. Save the R-file "MOI-MLE-IDM.R" in a directory `path` and load it using the function `source`. E.g., if the file is stored in source `"C:/Documents/backslash/Musterfrau"`, the file is loaded by running the following line.

```
source("C:/Documents/backslash/Musterfrau/MOI-MLE-IDM.R")
```

Importing data using `DatImp`. Import molecular data using the function `DatImp(path)`. Here, `path` is the location where the molecular dataset is stored. Data needs to be stored in a standardized fashion (see section Data format) as either an ".xlsx"-, ".csv"- or ".txt"-file. If the data is stored in an ".xls"-file, it has to be converted into an ".xlsx"-file with appropriate spreadsheet software.

Code Example. The following code imports the file "STR.xlsx" (see additional files), which is stored in "C:/Documents/backslash/Molecular Data/STR.xlsx". Only the first 10 lines of output are shown.

```
path <- "C:/Documents/backslash/Molecular Data/STR.xlsx"
```

```
DatImp(path)
```

```
##         ID marker
## 1   MCP001    132
## 2     <NA>    144
## 3   MCP002    132
## 4     <NA>    126
## 5   MCP003    180
## 6   MCP003    144
## 7   MCP004     NA
## 8   MCP005    144
## 9   MCP006     NA
```

To read the data into an array named `dat` use the following code.

```
dat <- DatImp(path)
```

Data format. Molecular data needs to be stored either as ".xlsx"-, ".csv"- or ".txt"-file in a specific format. Examples are provided as additional files. The format for ".xlsx"-files is described. A data set consists of two columns. The first contains the sample IDs, the second molecular information from samples. Each sample is stored in a $2 \times k$ block. In the first column at least the first row must contain the sample ID. The lineages present in the sample are stored in the second column in consecutive rows in any arbitrary order. Below are four alternative schematic descriptions of a sample in which lineages 1, 2 and 4 were observed. Note that missing values can occur, that the same lineage might be entered multiple times for a sample (but it is counted only once) and that the sample ID has to occur only in the first row. Missing values must be left empty. Examples:

ID1	lineage 2
	lineage 4
	lineage 1

ID1	lineage 1
ID1	lineage 2
	lineage 4

ID1	lineage 1
	lineage 2
	lineage 4
	lineage 4

ID1	lineage 1
	lineage 2
	lineage 4
ID1	lineage 4

Sample IDs and lineages are entered as numbers or strings. See the additional file "STR.xlsx" for an example of microsatellite data and "SNP.xlsx" for SNP data. The first row is reserved for column labels. It can be left empty, but this row must not be omitted. The table below shows the first 10 rows of the example data set STR.xlsx, corresponding to the first 6 samples.

```
dat
```

```
##          ID marker
## 1   MCP001    132
## 2     <NA>    144
## 3   MCP002    132
## 4     <NA>    126
## 5   MCP003    180
## 6   MCP003    144
## 7   MCP004     NA
## 8   MCP005    144
## 9   MCP006     NA
## 10    <NA>    132
```

The first sample (MCP001) contains two lineages, "132" and "144". (The numbers corresponding to repeat lengths of the STR). The second sample (MCP002) lineages "132", "126"- Sample MCP003 contains the lineages "144" and "180". Note that the sample ID is entered in both rows for sample MCP003 but only in the first row for samples MCP001 and MCP002. Sample MCP004 is an empty record. Sample MCP005 contains only lineage "144", and sample MCP006 only lineage "132". However, sample MCP006 is entered in an awkward way.

If the data is stored as a ".txt"-file, columns have to be separated by a tab stop. If it is entered as a ".csv" file, columns have to be separated by a semicolon (examples are found as additional files).

Frequency counts using the function `Nk`. The function `Nk(dat)` takes a $2 \times s$ array containing the molecular data and yields sample size (including the empty records), lineage-frequency counts, and the number of empty records as a list object. The first list element is the sample size N, the second a matrix with the frequency counts $N_1, \ldots, N_n$, and the third is the number of empty records $n_\mathbf{0}$. The column names of the matrix are the respective lineages.

Code example. The following code takes the array `dat` corresponding to the sample data set "STR.xlsx" (see additional files) and calculates sample size, lineage frequency counts, and the number of empty records.

```
Nk(dat)
```

```
## $N
## [1] 99
##
## $N_k
##      126 132 144 150 180
## [1,]  22  25  49  32  18
##
## $n_0
## [1] 2
```

The data contains $N = 99$ samples. Five different STR repeats (lineages) are found in the data, namely, 126, 132, 144, 150, and 180. Their respective counts are $N_1 = 22$, $N_2 = 25$, $N_3 = 49$, $N_4 = 32$, and $N_5 = 18$. Two of samples (MCP004 and MCP008) are empty records, i.e., the number of empty records is $n_{\mathbf{0}} = 2$.

The function MLE. The function `MLE(N, N_k, n_0 = 0, model = "IDM", lambda_initial = 1, eps_initial = 0.1)` calculates the maximum likelihood estimate (MLE) $(\hat{\lambda}, \hat{p}_1, \ldots, \hat{p}_n)$ from the data N, $(N_1, \ldots, N_n)$, and $n_{\mathbf{0}}$ based on the IDM or OM. Note that the number of empty records, $n_{\mathbf{0}}$, is an optional argument (default `n_0 = 0`), which should be specified only if the data contains empty records. The function has the following optional arguments. The argument "model" specifies whether the IDM (`model = 'IDM'`; default), or the original model (`model = 'OM'`) is used. If the option is set to `model = 'OM'` the argument $n_{\mathbf{0}}$ can be omitted. A further argument is `lambda_initial` (default `lambda_initial = 1`), the initial value for the numerical iteration to find the estimate $\hat{\lambda}$. The default value can be changed to optimize computational time. Unless numerical problem occur, the default parameter should be used. Similarly, the argument `eps_initial` (default `eps_initial = 0.1`) specifies the initial value in the numerical iteration to find $\hat{\varepsilon}$.

The output is a list containing six elements: (1) the MLE of the probability of lineages being undetected $\hat{\varepsilon}$, (2) the MLE $\hat{\lambda}$ of MOI parameter, (3) the MLE of the average MOI $\hat{\psi}$, (4) the estimated frequencies $(\hat{p}_1, \ldots, \hat{p}_n)$, (5) the inverse Fisher information estimated at the MLE, which is an estimate for the covariance of the estimator, and (6) the inverse Fisher information adjusted for the average MOI, i.e., the covariance matrix for the parameters $(\hat{\psi}, \hat{\varepsilon}, \hat{p}_1, \ldots, \hat{p}_n)$. The first list element is omitted if `model = 'OM'`. (Note the inverse Fisher information and the inverse observed information coincide if evaluated at the MLE.)

Code example. This code calculates the MLE for data consisting of $N = 99$ samples with frequency counts $N_1 = 22$, $N_2 = 25$, $N_3 = 49$, $N_4 = 32$, and $N_5 = 18$, as well as $n_{\mathbf{0}} = 2$ empty records.

```
MLE(99, c(22,25,49,32,18), n_0 = 2)
```

```
## [1] "TEST"

## $`probability of lineages remain undetected`
## [1] 0.03411416
##
## $`MOI parameter lambda`
## [1] 1.269117
##
## $`average MOI`
## [1] 1.76531
##
## $`lineage frequencies`
## [1] 0.1424659 0.1640532 0.3620580 0.2168498 0.1145731
##
## $`inverse Fisher information`
##               lam           eps           p.1           p.2           p.3
## lam  3.062854e-02  1.325123e-03 -2.189894e-04 -1.993795e-04  7.287220e-04
```

```
## eps  1.325123e-03  5.822136e-04 -4.885643e-06 -4.278664e-06  1.570661e-05
## p.1 -2.189894e-04 -4.885643e-06  8.074933e-04 -1.430387e-04 -3.710921e-04
## p.2 -1.993795e-04 -4.278664e-06 -1.430387e-04  9.188075e-04 -4.323251e-04
## p.3  7.287220e-04  1.570661e-05 -3.710921e-04 -4.323251e-04  1.685543e-03
## p.4 -8.756611e-05 -1.384952e-06 -1.974393e-04 -2.309168e-04 -5.881128e-04
## p.5 -2.227869e-04 -5.157345e-06 -9.592312e-05 -1.125269e-04 -2.940125e-04
##               p.4           p.5
## lam -8.756611e-05 -2.227869e-04
## eps -1.384952e-06 -5.157345e-06
## p.1 -1.974393e-04 -9.592312e-05
## p.2 -2.309168e-04 -1.125269e-04
## p.3 -5.881128e-04 -2.940125e-04
## p.4  1.172104e-03 -1.556355e-04
## p.5 -1.556355e-04  6.580981e-04
##
## $`inverse Fisher information adjusted for average MOI`
##               psi           eps           p.1           p.2           p.3
## psi  2.146397e-02  9.286238e-04 -1.534641e-04 -1.397218e-04  5.106761e-04
## eps  9.286238e-04  5.822136e-04 -4.885643e-06 -4.278664e-06  1.570661e-05
## p.1 -1.534641e-04 -4.885643e-06  8.074933e-04 -1.430387e-04 -3.710921e-04
## p.2 -1.397218e-04 -4.278664e-06 -1.430387e-04  9.188075e-04 -4.323251e-04
## p.3  5.106761e-04  1.570661e-05 -3.710921e-04 -4.323251e-04  1.685543e-03
## p.4 -6.136485e-05 -1.384952e-06 -1.974393e-04 -2.309168e-04 -5.881128e-04
## p.5 -1.561253e-04 -5.157345e-06 -9.592312e-05 -1.125269e-04 -2.940125e-04
##               p.4           p.5
## psi -6.136485e-05 -1.561253e-04
## eps -1.384952e-06 -5.157345e-06
## p.1 -1.974393e-04 -9.592312e-05
## p.2 -2.309168e-04 -1.125269e-04
## p.3 -5.881128e-04 -2.940125e-04
## p.4  1.172104e-03 -1.556355e-04
## p.5 -1.556355e-04  6.580981e-04
```

The resulting estimates are $\hat{\varepsilon} = 0.03411416$, $\hat{\lambda} = 1.269117$, $\hat{\psi} = 1.76531$, $\hat{p}_1 = 0.1424659$, $\hat{p}_2 = 0.1640532$, $\hat{p}_3 = 0.3620580$, $\hat{p}_4 = 02168498$, and $\hat{p}_5 = 0.1145731$.

Code example. The above example corresponded to the data "STR.xlsx". The following two lines are an alternative syntax to calculate the MLE.

```
nk <- Nk(dat)
MLE(nk[[1]], nk[[2]], nk[[3]], model = "IDM")
```

```
## [1] "TEST"
```

```
## $`probability of lineages remain undetected`
## [1] 0.03411416
##
## $`MOI parameter lambda`
## [1] 1.269117
##
## $`average MOI`
## [1] 1.76531
##
## $`lineage frequencies`
## [1] 0.1424659 0.1640532 0.3620580 0.2168498 0.1145731
##
```

```
## $`inverse Fisher information`
##               lam           eps           p.1           p.2           p.3
## lam  3.062854e-02  1.325123e-03 -2.189894e-04 -1.993795e-04  7.287220e-04
## eps  1.325123e-03  5.822136e-04 -4.885643e-06 -4.278664e-06  1.570661e-05
## p.1 -2.189894e-04 -4.885643e-06  8.074933e-04 -1.430387e-04 -3.710921e-04
## p.2 -1.993795e-04 -4.278664e-06 -1.430387e-04  9.188075e-04 -4.323251e-04
## p.3  7.287220e-04  1.570661e-05 -3.710921e-04 -4.323251e-04  1.685543e-03
## p.4 -8.756611e-05 -1.384952e-06 -1.974393e-04 -2.309168e-04 -5.881128e-04
## p.5 -2.227869e-04 -5.157345e-06 -9.592312e-05 -1.125269e-04 -2.940125e-04
##               p.4           p.5
## lam -8.756611e-05 -2.227869e-04
## eps -1.384952e-06 -5.157345e-06
## p.1 -1.974393e-04 -9.592312e-05
## p.2 -2.309168e-04 -1.125269e-04
## p.3 -5.881128e-04 -2.940125e-04
## p.4  1.172104e-03 -1.556355e-04
## p.5 -1.556355e-04  6.580981e-04
##
## $`inverse Fisher information adjusted for average MOI`
##               psi           eps           p.1           p.2           p.3
## psi  2.146397e-02  9.286238e-04 -1.534641e-04 -1.397218e-04  5.106761e-04
## eps  9.286238e-04  5.822136e-04 -4.885643e-06 -4.278664e-06  1.570661e-05
## p.1 -1.534641e-04 -4.885643e-06  8.074933e-04 -1.430387e-04 -3.710921e-04
## p.2 -1.397218e-04 -4.278664e-06 -1.430387e-04  9.188075e-04 -4.323251e-04
## p.3  5.106761e-04  1.570661e-05 -3.710921e-04 -4.323251e-04  1.685543e-03
## p.4 -6.136485e-05 -1.384952e-06 -1.974393e-04 -2.309168e-04 -5.881128e-04
## p.5 -1.561253e-04 -5.157345e-06 -9.592312e-05 -1.125269e-04 -2.940125e-04
##               p.4           p.5
## psi -6.136485e-05 -1.561253e-04
## eps -1.384952e-06 -5.157345e-06
## p.1 -1.974393e-04 -9.592312e-05
## p.2 -2.309168e-04 -1.125269e-04
## p.3 -5.881128e-04 -2.940125e-04
## p.4  1.172104e-03 -1.556355e-04
## p.5 -1.556355e-04  6.580981e-04
```

Code example. This code calculates the MLE for the data “STR.xlsx” from the above examples using the original model.

```
MLE(99, c(22,25,49,32,18), n_0 = 2, model = "OM")
```

```
## [1] "Option model= `OM' neglects n_0=2 samples and adjusts sample size to N=97"
```

```
## $`MOI parameter lambda`
## [1] 1.218736
##
## $`average MOI`
## [1] 1.730185
##
## $`lineage frequencies`
## [1] 0.1428264 0.1643813 0.3608585 0.2169937 0.1149401
##
## $`inverse Fisher information`
##               lam           p.1           p.2           p.3           p.4
## lam  2.540442e-02 -1.810303e-04 -0.0001644661  0.0006013736 -7.129375e-05
## p.1 -1.810303e-04  8.061818e-04 -0.0001443005 -0.0003665538 -1.980275e-04
```

```
## p.2 -1.644661e-04 -1.443005e-04  0.0009158232 -0.0004265196 -2.311522e-04
## p.3  6.013736e-04 -3.665538e-04 -0.0004265196  0.0016624126 -5.784725e-04
## p.4 -7.129375e-05 -1.980275e-04 -0.0002311522 -0.0005784725  1.164144e-03
## p.5 -1.845835e-04 -9.730005e-05 -0.0001138508 -0.0002908666 -1.564922e-04
##               p.5
## lam -1.845835e-04
## p.1 -9.730005e-05
## p.2 -1.138508e-04
## p.3 -2.908666e-04
## p.4 -1.564922e-04
## p.5  6.585098e-04
##
## $`inverse Fisher information adjusted for average MOI`
##               psi           p.1           p.2           p.3           p.4
## psi  1.761985e-02 -1.255579e-04 -0.0001140694  0.0004170972 -4.944751e-05
## p.1 -1.255579e-04  8.061818e-04 -0.0001443005 -0.0003665538 -1.980275e-04
## p.2 -1.140694e-04 -1.443005e-04  0.0009158232 -0.0004265196 -2.311522e-04
## p.3  4.170972e-04 -3.665538e-04 -0.0004265196  0.0016624126 -5.784725e-04
## p.4 -4.944751e-05 -1.980275e-04 -0.0002311522 -0.0005784725  1.164144e-03
## p.5 -1.280223e-04 -9.730005e-05 -0.0001138508 -0.0002908666 -1.564922e-04
##               p.5
## psi -1.280223e-04
## p.1 -9.730005e-05
## p.2 -1.138508e-04
## p.3 -2.908666e-04
## p.4 -1.564922e-04
## p.5  6.585098e-04
```

Here, $\hat{\lambda} = 1.218736$, $\hat{\psi} = 1.730185$, $\hat{p}_1 = 0.1428264$, $\hat{p}_2 = 0.1643813$, $\hat{p}_3 = 0.3608585$, $\hat{p}_4 = 0.2169937$, and $\hat{p}_5 = 0.1149401$.

An alternative syntax are the following two lines.

```
nk <- Nk(dat)
MLE(nk[[1]], nk[[2]], nk[[3]], model = "OM")
```

```
## [1] "Option model= `OM' neglects n_0=2 samples and adjusts sample size to N=97"

## $`MOI parameter lambda`
## [1] 1.218736
##
## $`average MOI`
## [1] 1.730185
##
## $`lineage frequencies`
## [1] 0.1428264 0.1643813 0.3608585 0.2169937 0.1149401
##
## $`inverse Fisher information`
##               lam           p.1           p.2           p.3           p.4
## lam  2.540442e-02 -1.810303e-04 -0.0001644661  0.0006013736 -7.129375e-05
## p.1 -1.810303e-04  8.061818e-04 -0.0001443005 -0.0003665538 -1.980275e-04
## p.2 -1.644661e-04 -1.443005e-04  0.0009158232 -0.0004265196 -2.311522e-04
## p.3  6.013736e-04 -3.665538e-04 -0.0004265196  0.0016624126 -5.784725e-04
## p.4 -7.129375e-05 -1.980275e-04 -0.0002311522 -0.0005784725  1.164144e-03
## p.5 -1.845835e-04 -9.730005e-05 -0.0001138508 -0.0002908666 -1.564922e-04
##               p.5
```

```
## lam -1.845835e-04
## p.1 -9.730005e-05
## p.2 -1.138508e-04
## p.3 -2.908666e-04
## p.4 -1.564922e-04
## p.5  6.585098e-04
##
## $`inverse Fisher information adjusted for average MOI`
##               psi           p.1           p.2           p.3           p.4
## psi  1.761985e-02 -1.255579e-04 -0.0001140694  0.0004170972 -4.944751e-05
## p.1 -1.255579e-04  8.061818e-04 -0.0001443005 -0.0003665538 -1.980275e-04
## p.2 -1.140694e-04 -1.443005e-04  0.0009158232 -0.0004265196 -2.311522e-04
## p.3  4.170972e-04 -3.665538e-04 -0.0004265196  0.0016624126 -5.784725e-04
## p.4 -4.944751e-05 -1.980275e-04 -0.0002311522 -0.0005784725  1.164144e-03
## p.5 -1.280223e-04 -9.730005e-05 -0.0001138508 -0.0002908666 -1.564922e-04
##               p.5
## psi -1.280223e-04
## p.1 -9.730005e-05
## p.2 -1.138508e-04
## p.3 -2.908666e-04
## p.4 -1.564922e-04
## p.5  6.585098e-04
```

The same output is produced by the following code, which omits the number of empty records $n_\mathbf{0}$, and adjusts the sample size.

```
MLE(97, c(22,25,49,32,18), model = "OM")
```

```
## $`MOI parameter lambda`
## [1] 1.218736
##
## $`average MOI`
## [1] 1.730185
##
## $`lineage frequencies`
## [1] 0.1428264 0.1643813 0.3608585 0.2169937 0.1149401
##
## $`inverse Fisher information`
##               lam           p.1           p.2           p.3           p.4
## lam  2.540442e-02 -1.810303e-04 -0.0001644661  0.0006013736 -7.129375e-05
## p.1 -1.810303e-04  8.061818e-04 -0.0001443005 -0.0003665538 -1.980275e-04
## p.2 -1.644661e-04 -1.443005e-04  0.0009158232 -0.0004265196 -2.311522e-04
## p.3  6.013736e-04 -3.665538e-04 -0.0004265196  0.0016624126 -5.784725e-04
## p.4 -7.129375e-05 -1.980275e-04 -0.0002311522 -0.0005784725  1.164144e-03
## p.5 -1.845835e-04 -9.730005e-05 -0.0001138508 -0.0002908666 -1.564922e-04
##               p.5
## lam -1.845835e-04
## p.1 -9.730005e-05
## p.2 -1.138508e-04
## p.3 -2.908666e-04
## p.4 -1.564922e-04
## p.5  6.585098e-04
##
## $`inverse Fisher information adjusted for average MOI`
##               psi           p.1           p.2           p.3           p.4
```

```
## psi  1.761985e-02 -1.255579e-04 -0.0001140694  0.0004170972 -4.944751e-05
## p.1 -1.255579e-04  8.061818e-04 -0.0001443005 -0.0003665538 -1.980275e-04
## p.2 -1.140694e-04 -1.443005e-04  0.0009158232 -0.0004265196 -2.311522e-04
## p.3  4.170972e-04 -3.665538e-04 -0.0004265196  0.0016624126 -5.784725e-04
## p.4 -4.944751e-05 -1.980275e-04 -0.0002311522 -0.0005784725  1.164144e-03
## p.5 -1.280223e-04 -9.730005e-05 -0.0001138508 -0.0002908666 -1.564922e-04
##               p.5
## psi -1.280223e-04
## p.1 -9.730005e-05
## p.2 -1.138508e-04
## p.3 -2.908666e-04
## p.4 -1.564922e-04
## p.5  6.585098e-04
```

Simulations to ascertain precision and accuracy goals

The function `cpoiss`. The function `cpoiss(lambda, n)` generates n random numbers from a conditional poisson distribution with parameter λ.

Code example. This code generates 10 random numbers from a conditional Poisson distribution with parameter $\lambda = 1.5$.

```
cpoiss(1.5, 10)
```

```
##  [1] 1 1 1 3 1 1 2 1 2 5
```

The function `mnom`. The function `mnom(M, p)` generates a random vector $(m_1, \ldots, m_n)$ from a multinomial distribution with parameters `M` and `p <- c(p_1,...,p_n)`. The argument `M` is either a positive integer or a vector of positive integers, $M = (M_1, \ldots, M_k)$, in which case the output is a $k \times n$ matrix, where the ith row $(m_{i1}, \ldots, m_{in})$ follows a multinomial distribution with parameters M_i and $\boldsymbol{p}$.

Code example. This code generates a multinomial random vector with parameters $M = 8$ and $\boldsymbol{p} = (\frac{1}{4}, \frac{1}{4}, \frac{1}{4}, \frac{1}{4})$.

```
mnom(8,c(0.25,0.25,0.25,0.25))
```

```
##      [,1] [,2] [,3] [,4]
## [1,]    2    3    1    2
```

Code example. This code generates a multinomial random vector with parameters $M = (8, 5, 6)$ and $\boldsymbol{p} = (\frac{1}{4}, \frac{1}{4}, \frac{1}{4}, \frac{1}{4})$.

```
mnom(c(8,5,6),c(0.25,0.25,0.25,0.25))
```

```
##      [,1] [,2] [,3] [,4]
## [1,]    3    0    5    0
## [2,]    1    2    1    1
## [3,]    1    2    1    2
```

Simulating a data set with complete information. A sample corresponds to a $0-1$ vector of length n indicating the absence and presence of n possible lineages in the sample. A dataset of sample size N is an $N \times n$ matrix with entries 0 and 1. Each row corresponds to one sample. A data set is generated using the functions `cpoiss`, `mnom`, and `sign`.

Code example. This code generates a data set of $N = 10$ samples, assuming that MOI follows a conditional Poisson distribution with parameter $\lambda = 1.5$ and lineage frequency distribution $\boldsymbol{p} = (\frac{1}{4}, \frac{1}{4}, \frac{1}{4}, \frac{1}{4})$, and stores it as `sim.dat`. An output of this data set is then generated.

```r
sim.dat <- sign(mnom(cpoiss(1.5,10),c(0.25,0.25,0.25,0.25)))
sim.dat
```

```
##       [,1] [,2] [,3] [,4]
##  [1,]    1    0    0    0
##  [2,]    1    0    1    1
##  [3,]    0    1    0    1
##  [4,]    0    1    0    1
##  [5,]    1    0    1    0
##  [6,]    0    1    0    0
##  [7,]    1    0    0    0
##  [8,]    1    0    0    0
##  [9,]    1    0    0    1
## [10,]    1    0    1    0
```

The function `IncompleteData`. To incorporate incomplete information into simulated data, the function `IncompleteData(data, eps)` can be applied to a dataset with complete information ($0-1$-matrix of dimension $N \times n$). The first argument specifies the data set, the second argument the probability of lineages remaining undetected. The output is a modified data set ($0-1$-matrix of dimension $N \times n$), in which some lineages might remain undetected.

Code example. This code modifies the dataset `sim.dat`generated in the previous example assuming that the probability of a lineage to remain undetected in a sample is $\varepsilon = 0.15$.

```r
IncompleteData(sim.dat, 0.15)
```

```
##       [,1] [,2] [,3] [,4]
##  [1,]    1    0    0    0
##  [2,]    1    0    1    1
##  [3,]    0    1    0    1
##  [4,]    0    1    0    1
##  [5,]    1    0    1    0
##  [6,]    0    1    0    0
##  [7,]    1    0    0    0
##  [8,]    1    0    0    0
##  [9,]    1    0    0    0
## [10,]    0    0    0    0
```
