# Supplementary material for: Estimating multiplicity of infection, allele frequencies, and prevalences accounting for incomplete data
Source: PLoS One. 2024 Mar 21;19(3):e0287161. doi: 10.1371/journal.pone.0287161 (PMC10956774; doi:10.1371/journal.pone.0287161)
Supplement: S3 Data — (PDF) [file pone.0287161.s004.pdf]

S4 Data from Cameroon and Kenya

Meraj Hashemi, Kristan A. Schneider

Cameroon data

Data from [1] from 166 samples collected in Yaoundé Cameroon from 2001-2002.

Cameroon, marker J6

Alleles:	200	188	192	194	180	182	198	108	166	184	168	178	186	159	174	190	176	172	189	196
N_k:	4	8	7	7	9	17	3	1	5	6	3	13	16	1	3	7	4	1	1	5
N:	166																			
n_0:	65																			

Cameroon, marker K6

Alleles:	261	232	239	248	234	218	227	230	242	246	226	215	269	224	244	213	256	250	265	259	254	228	157	236	276	222	209	252	219	220	263
N_k:	3	11	8	8	20	3	2	10	6	9	8	1	1	4	10	3	3	4	1	11	2	6	1	2	1	1	1	4	1	1	1
N:	166																														
n_0:	46																														

Cameroon, marker U5

Alleles:	136	99	117	105	107	113	119	109	125	134	115	129	103	111	121	123	131	151	127	140	154	142	144
N_k:	3	5	13	6	15	16	5	7	4	3	9	5	4	11	9	3	4	1	7	1	1	1	1
N:	166																						
n_0:	56																						

Cameroon, marker U6

Alleles:	99	75	91	104	93	116	108	107	118	103	102	112	136	97	84	87	89	126	111	100	85	123	95	119	106	83	132	79	81	122
N_k:	16	2	6	16	12	4	10	11	7	12	16	8	1	12	2	7	7	2	6	7	2	1	4	3	2	2	1	2	1	2
N:	166																													
n_0:	16																													

Cameroon, marker L4

Alleles:	123	117	131	138	111	119	130	113	121	109	125	128	132	140	151	115	134	142	137	100	105	127	101	154	103	107
N_k:	17	7	3	2	5	10	10	7	8	3	9	4	7	3	1	4	1	2	5	1	1	3	1	1	1	5
N:	166																									
n_0:	61																									

Cameroon, marker L5

Alleles:	153	149	151	144	147	138	142	136	130	128	157	148	160	159	132	164	155	140	172	171	134	184	162	109
N_k:	17	9	11	17	12	10	14	6	7	2	1	5	3	1	2	1	3	6	1	1	1	1	1	1
N:	166																							
n_0:	50																							

Cameroon, marker J3

Alleles:	192	124	134	140	150	149	145	143	128	151	138	172	156	167	152	164	126	130	147	154
N_k:	2	47	3	21	4	5	4	8	3	2	8	2	2	1	2	1	5	1	3	1
N:	166																			
n_0:	56																			

Cameroon, marker L1

Alleles:	137	157	138	167	175	166	158	161	155	141	151	153	154	156	160	169	179	105	139	184	134	150	176	170	123
N_k:	7	26	27	14	2	8	32	4	1	8	3	1	11	1	2	2	1	1	4	1	1	1	2	2	1
N:	166																								
n_0:	47																								

Cameroon, marker E1

Alleles:	162	134	151	147	155	145	157	149	166	153	160	170	164	141	175	109	143	177	139
N_k:	40	1	7	6	6	3	4	15	2	5	3	1	3	1	3	1	2	1	1
N:	166																		
n_0:	69																		

Cameroon, marker FR10

Alleles:	265	259	256	270	253	262	264
N_k:	4	102	28	1	3	40	3
N:	166						
n_0:	17						

Cameroon, marker O1

Alleles:	285	283	287	292	281	289	290	278	294	296	272	302	279	300
N_k:	14	15	60	16	30	9	1	14	7	6	2	1	1	2
N:	166													
n_0:	22													

Cameroon, marker Q4

Alleles:	167	181	203	205	171	190	209	208	199	185	176	173	200	175	215	183	177	179	187	207	191	201	182	211	194	212	184	174	170	165	224	219	214
N_k:	12	8	25	17	7	2	3	3	3	4	2	9	16	7	1	10	2	11	1	4	4	6	1	3	1	1	1	1	1	1	1	1	1
N:	166																																
n_0:	32																																

Cameroon, marker C3

Alleles:	126	103	150	152	138	136	122	140	156	154	130	132	145	143	161	134	148	164	124	167	157	15	172	135	119
N_k:	6	1	8	32	12	7	2	3	4	4	3	16	5	3	2	10	1	2	5	1	1	1	1	1	1
N:	166																								
n_0:	54																								

Cameroon, marker FR9

Alleles:	216	226	222	219	256	229	235	232	228	213
N_k:	14	93	38	23	2	7	2	5	2	1
N:	166									
n_0:	15									

Kenya, marker U5

Alleles:	95	130	99	142	103	113	108	115	97	111	109	119	136	123	117	101	105	125	107	121
N_k:	2	2	8	1	2	2	1	3	4	3	3	4	1	3	2	1	7	1	3	2
N:	43																			
n_0:	0																			

Data from Kenya

Data from Asembo Bay, Kenya, collected between April 1993 and March 1994 from [2].

Kenya, marker K6

Alleles:	224	240	248	207	246	220	230	210	242	252	218	236	254	226	244	238	216	212	255	234	228	263	250	214	222	249
N_k:	3	6	3	1	2	6	5	3	6	2	2	1	2	2	1	2	1	2	1	4	2	1	1	1	2	1
N:	43																									
n_0:	0																									

Kenya, marker J6

Alleles:	172	178	176	187	188	185	174	180	191	166	170	182	198	194	193
N_k:	2	3	4	4	4	6	5	9	4	1	3	4	1	1	1
N:	43														
n_0:	1														

Kenya, marker U6

Alleles:	93	91	107	103	105	94	97	99	111	117	102	87	113	122
N_k:	4	6	8	5	3	6	5	5	5	1	1	3	1	1
N:	43													
n_0:	0													

Kenya, marker U7

Alleles:	210	208	212	204	211	213	214	209	207
N_k:	18	11	3	1	2	1	2	3	1
N:	43								
n_0:	2								

Kenya, marker L4

Alleles:	109	115	125	128	130	126	113	141	127	121	132	145	117	143	119	105	123	139
N_k:	4	9	4	4	2	1	4	3	1	8	3	1	3	2	2	2	2	1
N:	43																	
n_0:	0																	

Kenya, marker L5

Alleles:	142	147	144	151	155	139	131	154	129	148	157	200	136	138	133	141
N_k:	9	8	6	6	2	5	4	1	1	1	3	1	2	1	5	1
N:	43															
n_0:	1															

Kenya, marker J3

Alleles:	138	132	140	143	155	124	151	122	149	147	134	146	136	148	130	145
N_k:	5	3	2	6	1	2	2	16	1	3	4	2	3	1	1	1
N:	43															
n_0:	1															

Kenya, marker L1

Alleles:	137	140	165	157	150	159	109	119	171	153	122	124	120	130	174	133
N_k:	13	5	9	11	3	4	1	2	3	4	1	1	1	2	2	2
N:	43															
n_0:	0															

References

[1] McCollum AM, Mueller K, Villegas L, Udhayakumar V, Escalante AA. Common origin and fixation of Plasmodium falciparum dhfr and dhps mutations associated with sulfadoxine-pyrimethamine resistance in a low-transmission area in South America. Antimicrobial agents and chemotherapy. 2007;51(6):2085–2091. doi:10.1128/AAC.01228-06.

[2] McCollum AM, Schneider KA, Griffing SM, Zhou Z, Kariuki S, Ter-Kuile F, et al. Differences in Selective Pressure on Dhps and Dhfr Drug Resistant Mutations in Western Kenya. Malaria Journal. 2012;11(1):77. doi:10.1186/1475-2875-11-77.
