# Supplementary material for: Estimating multiplicity of infection, allele frequencies, and prevalences accounting for incomplete data
Source: PLoS One. 2024 Mar 21;19(3):e0287161. doi: 10.1371/journal.pone.0287161 (PMC10956774; doi:10.1371/journal.pone.0287161)
Supplement: Peer review history

# Peer review: Decision Letter 0

**DOI:** 10.1371/journal.pone.0287161.r001

**Authors:** Joshua Nosanchuk

6 Jun 2023

Dear Professor Wahyuningsih,

(Hi Retno! Nice paper! I hope that the revisions will not be too difficult! Josh)

Thank you very much for submitting your manuscript "The Seroprevalence of Anti-Histoplasma capsulatum IgG Antibody among Pulmonary Tuberculosis Patients in Seven Referral Tuberculosis Hospitals in Indonesia" for consideration at PLOS Neglected Tropical Diseases. As with all papers reviewed by the journal, your manuscript was reviewed by members of the editorial board and by several independent reviewers. In light of the reviews (below this email), we would like to invite the resubmission of a significantly-revised version that takes into account the reviewers' comments. In particular, the issue raised about performing "inferential statistics" should be addressed.

We cannot make any decision about publication until we have seen the revised manuscript and your response to the reviewers' comments. Your revised manuscript is also likely to be sent to reviewers for further evaluation.

When you are ready to resubmit, please upload the following:

[1] A letter containing a detailed list of your responses to the review comments and a description of the changes you have made in the manuscript. Please note while forming your response, if your article is accepted, you may have the opportunity to make the peer review history publicly available. The record will include editor decision letters (with reviews) and your responses to reviewer comments. If eligible, we will contact you to opt in or out.

[2] Two versions of the revised manuscript: one with either highlights or tracked changes denoting where the text has been changed; the other a clean version (uploaded as the manuscript file). 

Important additional instructions are given below your reviewer comments.

Please prepare and submit your revised manuscript within 60 days. If you anticipate any delay, please let us know the expected resubmission date by replying to this email. Please note that revised manuscripts received after the 60-day due date may require evaluation and peer review similar to newly submitted manuscripts.

Thank you again for your submission. We hope that our editorial process has been constructive so far, and we welcome your feedback at any time. Please don't hesitate to contact us if you have any questions or comments.

Sincerely,

Joshua Nosanchuk, MD

Section Editor

PLOS Neglected Tropical Diseases

***********************

Reviewer's Responses to Questions

**Key Review Criteria Required for Acceptance?**

As you describe the new analyses required for acceptance, please consider the following:

**Methods**

-Are the objectives of the study clearly articulated with a clear testable hypothesis stated?

-Is the study design appropriate to address the stated objectives?

-Is the population clearly described and appropriate for the hypothesis being tested?

-Is the sample size sufficient to ensure adequate power to address the hypothesis being tested?

-Were correct statistical analysis used to support conclusions?

-Are there concerns about ethical or regulatory requirements being met?

Reviewer #1: (No Response)

Reviewer #2: -Yes, objectives are clearly stated and testable

-Yes, the study design is appropriate

-Yes, the population is clearly described and appropriate though it would have been interesting to have a group of asymptomatic participants

-Yes, the sample size is sufficient

-Yes, correct statistical analysis was used. But testing for possible associated risk factors may add more value to the manuscript

-No, concerns

--------------------

**Results**

-Does the analysis presented match the analysis plan?

-Are the results clearly and completely presented?

-Are the figures (Tables, Images) of sufficient quality for clarity?

Reviewer #1: (No Response)

Reviewer #2: -Yes, the analysis presented match the plan

-Yes, results are clearly presented

-yes, figures are of sufficient quality and clarity

--------------------

**Conclusions**

-Are the conclusions supported by the data presented?

-Are the limitations of analysis clearly described?

-Do the authors discuss how these data can be helpful to advance our understanding of the topic under study?

-Is public health relevance addressed?

Reviewer #1: (No Response)

Reviewer #2: -Yes, conclusions are supported by the data

-Yes, limitations are clearly described

-Yes, the authors do explain how the results will advance research and care of suspected TB patients

-Yes it is

--------------------

**Editorial and Data Presentation Modifications?**

Use this section for editorial suggestions as well as relatively minor modifications of existing data that would enhance clarity. If the only modifications needed are minor and/or editorial, you may wish to recommend “Minor Revision” or “Accept”. 

Reviewer #1: (No Response)

Reviewer #2: The work is of health importance. However, some worries need to be addressed.

Why was Histoplasma antigen not also tested here as it may have help in disease classification?

I expect the study population to be "presumptive pulmonary TB patients" and not pulmonary TB patients as one of your study group is made of not microbiological confirmed PTB patients. please modify all over the manuscript.

line 75: From "from other...to malignancies", please rephrase for clarity

Line 104: use another word to replace countries. You may use "setting", "area" ...or remove Africa and cite instead some African countries.

Line 224: from "our findinds" to "Indonesia", please rephrase for better understanding.

--------------------

**Summary and General Comments**

Use this section to provide overall comments, discuss strengths/weaknesses of the study, novelty, significance, general execution and scholarship. You may also include additional comments for the author, including concerns about dual publication, research ethics, or publication ethics. If requesting major revision, please articulate the new experiments that are needed.

Reviewer #1: This is a nice study from the team from Indonesia.

The group found a relatively high prevalence of anti-histoplasma antibody titres in some patients.

However, the manuscript doesn't answer the question "Who should be screened ?"

I would suggest that the authors run inferential statistics to answer the above questions, which is of epidemiologic significance.

There are so many people with TB, histoplasmosis is rare, so who should we screen ?

Reviewer #2: (No Response)

--------------------

PLOS authors have the option to publish the peer review history of their article (what does this mean?). If published, this will include your full peer review and any attached files.

If you choose “no”, your identity will remain anonymous but your review may still be made public.

**Do you want your identity to be public for this peer review?** For information about this choice, including consent withdrawal, please see our Privacy Policy.

Reviewer #1: No

Reviewer #2: No

Figure Files:

While revising your submission, please upload your figure files to the Preflight Analysis and Conversion Engine (PACE) digital diagnostic tool, https://pacev2.apexcovantage.com. PACE helps ensure that figures meet PLOS requirements. To use PACE, you must first register as a user. Then, login and navigate to the UPLOAD tab, where you will find detailed instructions on how to use the tool. If you encounter any issues or have any questions when using PACE, please email us at figures@plos.org.

Data Requirements:

Please note that, as a condition of publication, PLOS' data policy requires that you make available all data used to draw the conclusions outlined in your manuscript. Data must be deposited in an appropriate repository, included within the body of the manuscript, or uploaded as supporting information. This includes all numerical values that were used to generate graphs, histograms etc.. For an example see here: http://www.plosbiology.org/article/info%3Adoi%2F10.1371%2Fjournal.pbio.1001908#s5.

Reproducibility:

To enhance the reproducibility of your results, we recommend that you deposit your laboratory protocols in protocols.io, where a protocol can be assigned its own identifier (DOI) such that it can be cited independently in the future. Additionally, PLOS ONE offers an option to publish peer-reviewed clinical study protocols. Read more information on sharing protocols at https://plos.org/protocols?utm_medium=editorial-email&utm_source=authorletters&utm_campaign=protocols

# Author comment: Author response to Decision Letter 0

**DOI:** 10.1371/journal.pone.0287161.r002

31 Jul 2023

Attachment Submitted filename: Letter_Histoplasmosis Response to Reviewers rev - edit.docx

# Editor report: Decision Letter 1

**DOI:** 10.1371/journal.pone.0287161.r003

**Authors:** Joshua Nosanchuk

6 Aug 2023

Dear Professor Wahyuningsih,

Thank you for the thoughtful and complete response to the reviewers' comments on your interesting manuscript. We are pleased to inform you that your manuscript 'The Seroprevalence of Anti-Histoplasma capsulatum IgG Antibody among Pulmonary Tuberculosis Patients in Seven Referral Tuberculosis Hospitals in Indonesia' has been provisionally accepted for publication in PLOS Neglected Tropical Diseases.

Before your manuscript can be formally accepted you will need to complete some formatting changes, which you will receive in a follow up email. A member of our team will be in touch with a set of requests.

Please note that your manuscript will not be scheduled for publication until you have made the required changes, so a swift response is appreciated.

IMPORTANT: The editorial review process is now complete. PLOS will only permit corrections to spelling, formatting or significant scientific errors from this point onwards. Requests for major changes, or any which affect the scientific understanding of your work, will cause delays to the publication date of your manuscript.

Should you, your institution's press office or the journal office choose to press release your paper, you will automatically be opted out of early publication. We ask that you notify us now if you or your institution is planning to press release the article. All press must be co-ordinated with PLOS.

Thank you again for supporting Open Access publishing; we are looking forward to publishing your work in PLOS Neglected Tropical Diseases.

Best regards,

Joshua Nosanchuk, MD

Section Editor

PLOS Neglected Tropical Diseases

***********************************************************

# Editor report: Acceptance letter

**DOI:** 10.1371/journal.pone.0287161.r004

**Authors:** Joshua Nosanchuk

6 Sep 2023

Dear Professor Wahyuningsih,

We are delighted to inform you that your manuscript, "The Seroprevalence of Anti-Histoplasma capsulatum IgG Antibody among Pulmonary Tuberculosis Patients in Seven Referral Tuberculosis Hospitals in Indonesia," has been formally accepted for publication in PLOS Neglected Tropical Diseases.

We have now passed your article onto the PLOS Production Department who will complete the rest of the publication process. All authors will receive a confirmation email upon publication.

The corresponding author will soon be receiving a typeset proof for review, to ensure errors have not been introduced during production. Please review the PDF proof of your manuscript carefully, as this is the last chance to correct any scientific or type-setting errors. Please note that major changes, or those which affect the scientific understanding of the work, will likely cause delays to the publication date of your manuscript. Note: Proofs for Front Matter articles (Editorial, Viewpoint, Symposium, Review, etc...) are generated on a different schedule and may not be made available as quickly.

Soon after your final files are uploaded, the early version of your manuscript will be published online unless you opted out of this process. The date of the early version will be your article's publication date. The final article will be published to the same URL, and all versions of the paper will be accessible to readers.

Thank you again for supporting open-access publishing; we are looking forward to publishing your work in PLOS Neglected Tropical Diseases. 

Best regards,

Shaden Kamhawi

co-Editor-in-Chief

PLOS Neglected Tropical Diseases

Paul Brindley

co-Editor-in-Chief

PLOS Neglected Tropical Diseases
